# Biocatalytic synthesis of 2′‐deoxynucleotide 5′‐triphosphates from bacterial genomic DNA: Proof of principle

**DOI:** 10.1002/bit.28374

**Published:** 2023-03-25

**Authors:** Anna R. Bird, Jennifer C. Molloy, Elizabeth A. H. Hall

**Affiliations:** ^1^ Chemical Engineering and Biotechnology University of Cambridge Cambridge UK

**Keywords:** dNTP synthesis, nucleic acid test reagents, nucleotide synthesis, on‐demand reagent synthesis

## Abstract

2′‐deoxynucleoside 5′‐triphosphates (dNTPs) are the building blocks of DNA and are key reagents which are incorporated by polymerase enzymes during nucleic acid amplification techniques, such as polymerase chain reaction (PCR). These techniques are of high importance, not only in molecular biology research, but also in molecular diagnostics. dNTPs are generally produced by a bottom‐up technique which relies on synthesis or isolation of purified small molecules like deoxynucleosides. However, the disproportionately high cost of dNTPs in low‐ and middle‐income countries (LMICs) and the requirement for cold chain storage during international shipping makes an adequate supply of these molecules challenging. To reduce supply chain dependency and promote domestic manufacturing in LMICs, a unique top‐down biocatalytic synthesis method is described to produce dNTPs. Readily available bacterial genomic DNA provides a crude source material to generate dNTPs and is extracted directly from *Escherichia coli* (step 1). Nuclease enzymes are then used to digest the genomic DNA creating monophosphorylated deoxynucleotides (dNMPs) (step 2). Design and recombinant production and characterization of *E. coli* nucleotide kinases is presented to further phosphorylate the monophosphorylated products to generate dNTPs (step 3). Direct use of the in‐house produced dNTPs in nucleic acid amplification is shown (step 4) and their successful use as reagents in the application of PCR, thereby providing proof of principle for the future development of recombinant nucleases and design of a recombinant solid‐state bioreactor for on‐demand dNTP production.

## Graphical abstract

2′‐deoxynucleoside 5′‐triphosphates (dNTPs), generated from genomic DNA (gDNA) are demonstrated as low‐cost DNA building blocks in nucleic acid amplification. gDNA was extracted from *Escherichia coli* and digested with nucleases to monophosphorylated deoxynucleotides (dNMPs). Recombinant *E. coli* nucleotide kinases were designed to phosphorylate the dNMPs to generate dNTPs.

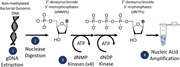

**Abbreviations**
adk: adenylate kinase from *E. coli*
ADP: adenosine 5′‐diphosphate
ATP: adenosine 5′‐triphosphate
Benz: benzonase
cmk: cytidylate kinase from *E. coli*
dAMP/dADP/dATP: 2′‐deoxyadenosine 5′‐mono/di/tri‐phosphate
dCMP/dCDP/dCTP: 2′‐deoxycytidine 5′‐mono/di/tri‐phosphate
dGMP/dGDP/dGTP: 2′‐deoxyguanosine 5′‐mono/di/tri‐phosphate
DNA: deoxyribonucleic acid
dNDP: 2′‐deoxynucleotide 5′‐diphosphate
dNMP: 2′‐deoxynucleotide 5′‐monophosphate
dNTP: 2′‐deoxynucleotide 5′‐triphosphate
dTMP/dTDP/dTTP: 2′‐deoxythymidine 5′‐mono/di/tri‐phosphate
ExoI: Exonuclease I from *E. coli*
ExoIII: Exonuclease III from *E. coli*
gDNA: genomic DNA
gmk: guanylate kinase from *E. coli*
HPLC: high pressure liquid chromatography
kb: kilobasepairs
LMIC: low‐ and middle‐income countries
MWCO: molecular weight cut‐off
NAAT: nucleic acid test
NADH: nicotinamide adenine dinucleotide
ndk: nucleotide disphosphate kinase from *E. coli*
NEB: New England Biolabs
PCR: polymerase Chain Reaction
PEP: phosphoenolpyruvate
POC: point‐of‐care
tmk: thymidylate kinase from *E. coli*

## 1 INTRODUCTION

Nucleic acid amplification tests (NAATs) have enabled a burgeoning of highly specific and sensitive diagnostics for infectious diseases and inherited or genetic‐linked biomarkers of chronic disease. Importantly it has become evident that use of NAATs can have a beneficial clinical impact on the patients (Reischl et al., 2020; Taegtmeyer et al., 2008; Wiersinga et al., 2009) by guiding treatment earlier than culture‐based methods or by identifying targeted therapies for certain cancer‐related mutations (Rowley et al., 2021; Simarro et al., 2022). Furthermore, the earlier diagnosis of infectious disease can have a significant impact on the stewardship of antimicrobial and antifungal resistance. It is reported, for example, that administration of unnecessary antifungal drugs could be reduced by >90% through reliable detection of *Candida* infections. Studies have also reported on the improved cost effectiveness of multiplexed PCR in the detection of infectious diseases, compared with other methods (Mahony et al., 2009).

Nevertheless, despite evidence of the benefits of early testing and the role that nucleic acid testing can play, there is a big gap in the capabilities of low‐ and middle‐income countries (LMICs), driven mainly by the high cost of NAATs, when they are imported from high‐income countries. Many African countries, for example, are only able to spend < $150 per capita on health, compared with high‐income countries spending > $4,000, so there is a fiscal as well as a facility barrier to the use of diagnostics (Africa Renewal, 2020). There is also widespread concern about the scale of imports and donor dependence across Africa. Growing attention is now being paid to local production capabilities for health technologies as a vital component of strengthening the health systems of developing countries. For example, the production of point‐of‐care (POC) diagnostics in LMICs has been proposed as essential for a technology fit for a low‐resource area and sustaining the supply chain (Engel et al., 2016). Numerous African government reports since 2000 have highlighted the potential for developmental synergies to be extracted between expansion of industrial production of medical supplies and improvement of the coverage and quality of health care, especially for their low‐income populations (MacKintosh et al., 2018). In May 2019, six international organizations with influence on global health (WHO, UNIDO, UNCTAD, UNAIDS, UNICEF, and The Global Fund) released a joint statement promoting local production of medicines and health technologies, laying out the need for “effective multisectoral collaboration to promote enabling investment, legal and technical environments” in these countries (World Health Organisation, 2019).

LMIC markets for PCR are expected to grow, but with revenue continuing to be generated outside the LMICs. However, a shift to local manufacture within many LMICs still has a cost disadvantage: their individual markets are comparatively small and most potential African manufacturers, for example, would have to import all their active reagents and materials for diagnostics in small quantities and at higher cost than competing overseas suppliers of the finished diagnostic; this same issue is also a barrier to local production of essential pharmaceuticals (Wilson et al., 2012). Thus, synergies between expansion of a local manufacturing infrastructure and the goal to achieve socioeconomic long‐term independence from external supply needs to include even the basic active reagents and materials.

Recently there have been reports of local production of some of the reagents required for NAATs. In earlier work, we have reported the local production of a self‐indicating mCherry fused BST_LF_ with a silica binding tag for immobilization, that was produced locally in Ghana for a loop‐mediated isothermal amplification (LAMP) for malaria diagnosis of clinical samples (Seevaratnam et al., 2022). The COVID‐19 pandemic has also shown that the enzymes for RT‐LAMP can be successfully produced in‐house locally without the need for import of these vital reagents (Matute et al., 2021) and Mote et al. have produced a PCR master mix with reagents produced in‐house (Mote et al., 2021).

Nevertheless, the building blocks for the synthesis of the amplified DNA, namely the 2′‐deoxynucleoside 5′‐triphosphates (dNTPs) remain the missing piece of the ‘local production' puzzle. These molecules are usually synthesized chemically (Burgess & Cook, 2000), which often lacks regioselectivity, thus resulting in the formation of unwanted products, reducing overall yield. A review paper published in 2016 included over 40 schemes on different methods to synthesize nucleotides and their analogs and concludes that “an ideal synthesis would involve little or no excess reagents and activators, short reaction times, the absence of protecting groups on the starting material, simple conditions (i.e., nondry solvents and reagents, open flasks, room temperature, etc.), high yields, and simple purification procedures” (Roy et al., 2016).

The high regioselectivity of enzyme‐catalyzed synthesis can ensure correct product formation and offer a greener alternative to harsh chemicals (Ahmad et al., 2009). Recent innovations include the synthesis of nucleotide analogs from modified nucleosides using a kinase and ATP regeneration system, and the in‐situ synthesis of dNTPs in recombinant *Escherichia coli* lysate (Fehlau et al., 2020; Loan et al., 2019). However, this does not eliminate supply chain dependency as the deoxynucleosides must still be purchased from chemical suppliers.

The dNTPs should be provided at concentrations that do not limit the reaction and, therefore must be employed in excess of other diagnostic reagents (Gibbs, 1990). A standard polymerase chain reaction (PCR) requires 20–200 µM dNTPs (Lorenz, 2012), but other nucleic acid amplification methods, such as loop‐mediated isothermal amplification (LAMP), that produce very long polymer amplicons, can require concentrations up to 1.5 mM. Furthermore, nucleoside triphosphates are not particularly robust, and their decomposition, which is believed to be caused by hydrolysis of the phosphate groups, demands that dNTPs are stored frozen, and thus proper cold chain storage must be maintained during shipping (Markoulatos et al., 2002). This presents an additional burden on access to these reagents and a study on challenges to good lab practices in East Africa found that many reagents were received with compromised cold chain storage (Zhang et al., 2016). Improvements have been made to the storage buffers which aids in long‐term stability at −20°C and prevent decomposition of dNTPs reported during multiple freeze‐thaw cycles (Loan et al., 2019). Some manufacturers now offer proprietary modified dNTPs (e.g., a thermolabile 3′‐tetrahydrofuranyl protecting group on the base which is released by heat) that offer greater stability at room temperature for up to a month (Le et al., 2009). However, for unmodified dNTPs in standard storage buffers, manufacturers generally recommend low‐temperature storage. In general, dNTPs are found to have time‐limited stability at ambient temperature and even at 4°C, whereas at higher temperatures the degradation rate increases: from other work from our group, this is significant above 60°C, in which a 10 mM solution of each dNTP (dATP, dCTP, dGTP, and dTTP) in amplification buffer has shown circa 7% per hour degradation (Gao et al., 2023). Thus, in low‐resource contexts where reliable consistent cold temperature storage is not always available, for example, due to power cuts, a method to produce dNTPs on‐demand can become critical. Additionally, prolonged exposure at ambient temperature during delays in shipping and customs could lead to degradation of dNTPs and failed application in nucleic acid amplification.

Thus, rather than starting from commercially available small molecules, Haynie and Whitesides produced nucleoside triphosphate products (NTPs) from RNA by digesting RNA from yeast with nuclease P1 and incubating the resulting NMP products with a mixture of nucleoside monophosphokinases and acetate kinase (Haynie & Whitesides, 1990).

The nuclease P1 belongs to a subfamily of phosphodiesterases that specifically degrade phosphodiester linkages in the nucleic acid backbone. Also among this subfamily are DNases, RNases, endonucleases, and exonucleases. The in vivo function of these enzymes is DNA repair, proofreading, and replication (Lovett, 2011), but they have also been leveraged commercially for applications such as DNA sequencing and PCR clean‐up. Phosphodiesterases from other organisms, including snake venom phosphodiesterase (SVP) and bovine spleen phosphodiesterase, have been used for the stepwise hydrolysis of DNA to release mononucleotides. 5′‐phosphodiesterase from malt root, for example, has produced 5′‐deoxynucleosides from denatured DNA (Zou et al., 2008), while SVP has been used in conjunction with phosphatases to prepare DNA samples for mass spectrometry analysis (Yin et al., 2018).

**Figure 1:**
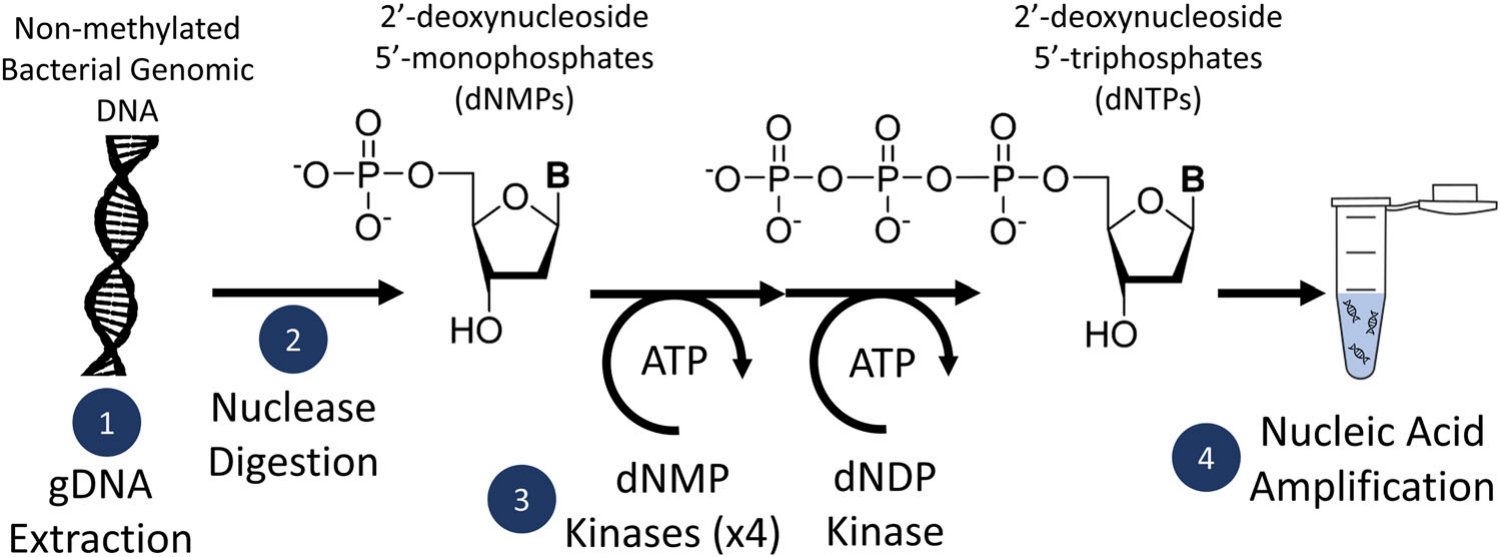
A novel method for biocatalytic synthesis of 2′‐deoxynucleotide 5′‐triphosphate (dNTPs) from bacterial genomic DNA. Non‐methylated genomic DNA (gDNA) is extracted from *Escherichia coli* JM110 (step 1). The gDNA is digested by nucleases (step 2), resulting in monophosphorylated nucleotides (2′‐deoxynucleotide 5′‐monophosphate [dNMPs]). The dNMPs are converted to nucleoside triphosphates (2′‐deoxynucleotide 5′‐triphosphate [dNTPs]) using recombinantly produced kinase proteins (step 3). The synthesized dNTPs can then be used directly in nucleic acid amplification (step 4).

In this manuscript, we take the next step and present a novel enzyme cascade to biocatalytically synthesize dNTPs from a low‐cost regenerable source, namely bacterial genomic DNA (Figure 1), and then use them in nucleic acid amplification. The bacteria starting material (*E. coli* JM110 in this example), provides the genomic DNA, which is then digested by phosphodiesterases to produce monophosphorylated nucleotide products (dNMPs). These are then rebuilt with kinases and ATP to produce dNTPs, which can be used directly in nucleic acid amplification.

## 2 MATERIALS AND METHODS

### 2.1 Materials

*E. coli* DH5α (NEB C2987), NEBTurbo (NEB C2984), Klenow (NEB M0210), BL21(DE3) (Agilent 200131), *E. coli* JM110 (Yanisch‐Perron et al., 1985), Ni‐NTA resin (Merck 69670), CentriPure P25 columns (emp Biotech Cat. No. CP‐0108), pyruvate kinase (Sigma P1506), lactate dehydrogenase (Sigma L2500), dAMP (Sigma D6375), dCMP (Sigma D7750), dGMP (Sigma D9500), dTMP (Sigma T7004), dADP (Sigma D600), dCDP (Cayman Chemicals 22982), dGDP (Sigma D2950), dTDP (Sigma T9375), NADH (Acros Organics 271100010), PEP (Cayman Chemicals 19192), ATP (Sgima A7699), ADP (Acros Organics 10143940), dNTP Set (NEB N0446), Monarch genomic DNA extraction kit (NEB T3010), 10 kDa MWCO columns (Merck 10125580), tetrabutylammonium phosphate (Fisher Scientific 10569092), ammonium dihydrogen phosphate (Sigma 101126), SeeBlue Plus2 pre‐stained protein ladder (ThermoFisher LC5925), NSR pre‐stained protein ladder (Newmarket Scientific MG20‐10101), 1 kb DNA ladder (NEB N3232), 1 kb plus DNA ladder (NEB N3200), HindIII‐HF (NEB R3104S), Exonuclease III (NEB M0206), benzonase (Sigma E1014).

### 2.2 Auto‐induction media

To prepare 4X YT media, 6 g of Na_2_HPO_4_, 3 g of KH_2_PO_4_, 20 g of tryptone, 5 g of yeast extract, and 5 g of NaCl were dissolved in 0.5 mL of DI water. The media was sterilized by autoclaving. 50% glycerol (v/v), 10% glucose (w/v), and 5% lactose (w/v) solutions were prepared separately and autoclaved. To prepare complete auto‐induction media, add 12 mL of 50% glycerol, 5 mL of 10% glucose, and 40 mL of 5% lactose to the 0.5 L of 4X YT media and bring the volume up to 1 L with sterile DI water (OpenWetWare Lidstrom: Autoinduction Media).

### 2.3 Protein expression and purification

Adenylate kinase (adk), cytidylate kinase (cmk), guanylate kinase (gmk), thymidylate kinase (tmk), and nucleotide disphosphate kinase (ndk) genes were amplified from the *E. coli* DH5α genome. Genes were assembled with a pET24a backbone including a C‐terminal 6x histidine tag and kanamycin resistance using Klenow assembly (OpenWetWare Klenow Assembly Method: Seamless cloning). Assembled plasmids were transformed into NEBTurbo for cloning, followed by transformation into BL21(DE3) for expression. Expression was performed by inoculating 50 mL of auto‐induction media with 0.5 mL of overnight culture and incubation overnight at 37°C with 225 rpm. Proteins were purified with Ni‐NTA resin according to the manufacturer's protocol, and the concentration was estimated with NanoDrop. Buffer exchange was performed with CentriPure P25 columns. Briefly, the column was equilibrated with 25 mL of 50 mM Tris‐HCl. Then, the purified protein eluted from the Ni‐NTA column was loaded onto the P25 column, followed by an additional 3.5 mL of 50 mM Tris‐HCl buffer. The eluate was collected in 0.5 mL fractions and the protein concentration of each was estimated using a NanoDrop A280 nm measurement. The fractions containing protein were combined, and the final purified proteins were stored in 25 mM Tris‐HCl, 50% glycerol at −20°C.

### 2.4 Enzyme kinetics

Enzyme kinetics were measured with the pyruvate kinase‐lactate dehydrogenase coupled spectrophotometric assay (Agarwal et al., 1978). The final reaction buffer consisted of 50 mM potassium acetate, 20 mM Tris‐acetate, 10 mM Magnesium acetate, 100 μg/mL BSA, 3 mM PEP, 2 mM ATP, 0.3 mM NADH, 1.25 U/mL pyruvate kinase, 2.25 U/mL lactate dehydrogenase, 0.0025 mg/mL protein, and varied concentration of substrate. Solutions were brought up to 37°C before adding the substrate. Absorbance at 340 nm was read immediately after addition of substrate and followed for 5–10 min with Tecan spark plate reader (Infinite M200). dNMP standards and dNDP standards in varying concentration were used as substrate.

### 2.5 Quantification of protein purity

The relative purity of the proteins was quantified by measuring the specific activity of total protein lysate relative to the specific activity of pure protein. The activity was measured as described above in the enzyme kinetics section. For the lysate samples, 0.0025 mg/mL of total protein (including native proteins and the kinase of interest) was used, whereas for pure samples, the 0.0025 mg/mL protein was entirely the protein of interest.

To test if native *E. coli* proteins interfered with the enzyme kinetics assay, a set of negative controls was performed using a BL21(DE3) lysate which did not contain overexpressed proteins. Five mL LB was inoculated with BL21(DE3) cells, which did not contain any plasmid, and incubated overnight at 37°C with 225 rpm. A 50 mL LB culture was inoculated with 0.5 mL of overnight culture and incubated for 6 h at 37°C with 225 rpm. The culture was pelleted to collect the cells, and the protein lysate was prepared as previously described. Enzyme kinetics experiments were performed against dAMP, dCMP, dGMP, dTMP, and dADP substrates using 0.0025 mg/mL BL21(DE3) lysate (Supporting Information: Figure S10).

### 2.6 Genomic DNA extraction and purification

*E. coli* JM110, which is *dam*‐ *dcm*‐, was obtained from AddGene (Yanisch‐Perron et al., 1985). *E. coli* JM110 was made competent with CCMB80 buffer (Hanahan et al., 1991; OpenWetWare TOP10 chemically competent cells) and was then transformed with a pET24a construct with Kanamycin resistance. Kanamycin‐resistant JM110 cells were cultured in 200 mL LB for 8 h. Cultures were pelleted in 50 mL aliquots by centrifugation at 3275 × *g* for 20 min. The supernatant was discarded, and the pellet was stored at −20°C until gDNA extraction. If cold chain is not available, the cells can be cultured and used immediately. Cell lysis was performed according to the simplified protocol for genomic DNA extraction from gram‐negative bacteria from NEB. Briefly, pellets were resuspended in 100 μL of PBS. Ten microliters of Proteinase K was added and the solution was briefly vortexed. Then, 100 μL of Tissue Lysis Buffer (NEB #T3010 proprietary composition) was added and the solution was vortexed thoroughly. The solution was incubated for 3–4 h in a shaking incubator at 56°C with 350 rpm. Next, 3 μL of RNase A was added to the lysate and the solution was incubated for 30 min at 56°C with 350 rpm.

To purify the DNA, 3 volumes of ice‐cold 100% EtOH (0.6 mL) was added to the lysate. The mixture was incubated on ice for 30 min. The lysate was centrifuged for 10 min at 13,000 rpm in a tabletop centrifuge at 4°C and the supernatant discarded. The pellet was washed three times with 0.5 mL of 70% EtOH and spun again at 13,000 rpm for 5 min at 4°C. The supernatant was discarded, and the pellet was airdried for 10 min. The pellet was resuspended in nuclease‐free water (50 µL), and the concentration was read with NanoDrop. We recognize that many cell lysate impurities absorb at 260 nm, and thus the NanoDrop reading is likely to overestimate the gDNA concentration. However, other methods, such as agarose gel analysis of concentration, are unreliable, so all yields are given from the measured NanoDrop concentration. If not required immediately, purified gDNA can be stored at −20°C until use.

### 2.7 In‐house dNTP synthesis

Concentrated gDNA was diluted in 50 mM potassium acetate, 20 mM Tris‐acetate, 10 mM Magnesium acetate, 100 μg/mL BSA, and combined with 100 units (U) of Exonuclease III and either 40 units restriction enzyme or 3.35 units of benzonase (enzyme units as recommended by manufacturer protocol). Where specified, 20 U of Exonuclease I was added to DNA digestion reactions. Water was added to a final reaction volume of 50 μL. Samples were incubated at 37°C for 16 h, then the nucleases were heat inactivated by incubating the samples at 80°C for 30 min. Next, in‐house produced NMP kinases were added to a final concentration of 0.004 mg/mL each and in‐house produced NDK was added to a final concentration of 0.008 mg/mL. 5 μL of 50 mM ATP was added. The solution was incubated at 37°C for 30 min, then used immediately in PCR reactions or prepared for analysis by HPLC. To prepare samples for HPLC, they were diluted 1:10 in water, then loaded onto a 10 kDa MWCO protein concentrator column and spun at 13,000 rpm in a tabletop centrifuge for 30 min. The filtrate was collected and filtered through a 0.2 μm syringe filter, then stored at −20⁰C until analysis.

Directly following the heat inactivate and kinase incubation. To concentrate the in‐house produced dNTPs, 5.56 μL of 3 M NaCl was added to the 50 μL reaction solution. Then, 165 μL of 100% ethanol was added, and the samples were incubated at −80°C or on ice for 1 h. The precipitated dNTPs were collected by centrifugation at 13,000 rpm in a benchtop centrifuge for 20 min. The supernatant was discarded, and the pellet was airdried for 30 min. The pellet was then resuspended in 18.75 μL of water and immediately used in PCR reactions (Caton‐Williams et al., 2011).

### 2.8 HPLC

Nucleotide products were analyzed by HPLC (Agilent Technologies 1260 Infinity II) following the method published by Loan et al. Briefly, samples were eluted from an Altima HP C18 column (5 μm, 250 × 4.6 mm HICH87680) by gradient elution (87%–70%) of buffer A. Buffer A consisted of aqueous 60 mM ammonium dihydrogen phosphate and 5 mM tetrabutylammonium phosphate at pH 5.0. Buffer B consisted of 5 mM tetrabutylammonium phosphate in methanol. Nucleotides were detected by monitoring at 259 nm (Loan et al., 2019). Analysis was performed over a 60‐min run time with 1 mL/min flow rate. The injection volume was 100 μL. Molecular identity of compounds was determined by comparison to the elution times of commercial standards diluted in 5 mM potassium acetate, 2 mM Tris‐acetate, 1 mM Magnesium acetate, 10 μg/mL BSA. Elution times are as follows: dCMP—4.52 ± 0.08 min, dGMP—6.86 ± 0.20 min, dTMP—7.98 ± 0.25 min, dAMP—13.14 ± 0.62 min, ADP—18.69 ± 0.07 min, dCTP—19.44 ± 0.29 min, dGTP—27.72 ± 0.26 min, dTTP—30.98 ± 0.36 min, ATP—34.56 ± 0.60 min, dATP—38.18 ± 0.60 min. Elution times shown are the average of *n* = 18 replicates for dNMP and dNTP standards and *n* = 3 replicates for ADP and ATP. The concentration of in‐house produced dNMPs and dNTPs was determined from a calibration curve of integration area versus concentration for each molecular standard (Supporting Information: Data S9–S12).

### 2.9 PCR

In‐house dNTPs were used in PCR reactions to amplify fragments from the lambda genome. Positive control reaction mix consisted of 1.25 μL 10 μM forward primer, 1.25 μL 10 μM reverse primer, 1 μL 50 μg/mL lambda genome DNA as template (NEB N3011), 2.5 μL 10X ThermoPol buffer, 16.75 μL water, 1.5 μL 100 mM MgSO_4_, 0.5 μL 10 mM commercial dNTP mix, and 0.25 μL of DeepVent polymerase. Negative controls lacked dNTPs. For PCR reactions using in‐house dNTPs, no commercial dNTPs or supplemental magnesium were added, and the water was replaced with 18.75 μL of dNTP solution. gDNA digestion negative control PCRs used 18.75 μL of gDNA digest solution (no kinases added) and did include 1 μL of template DNA (Supporting Information: Table S9). Thermocycling was as follows: 5 min of denaturation at 95°C, 31 cycles of 95°C for 30 s, 62°C for 30 s, and 72°C for the extension time listed in Supporting Information: Table S8, and a final extension at 72°C for 2 min.

In‐house dNTPs were in a buffer solution of 50 mM potassium acetate, 20 mM Tris‐acetate, 10 mM Magnesium acetate, 100 μg/mL BSA. This buffer composition was incompatible with Q5 Polymerase but did not interfere with DeepVent Polymerase reactions (data not shown).

## 3 RESULTS AND DISCUSSION

### 3.1 Genomic DNA extraction and digestion

**Figure 2:**
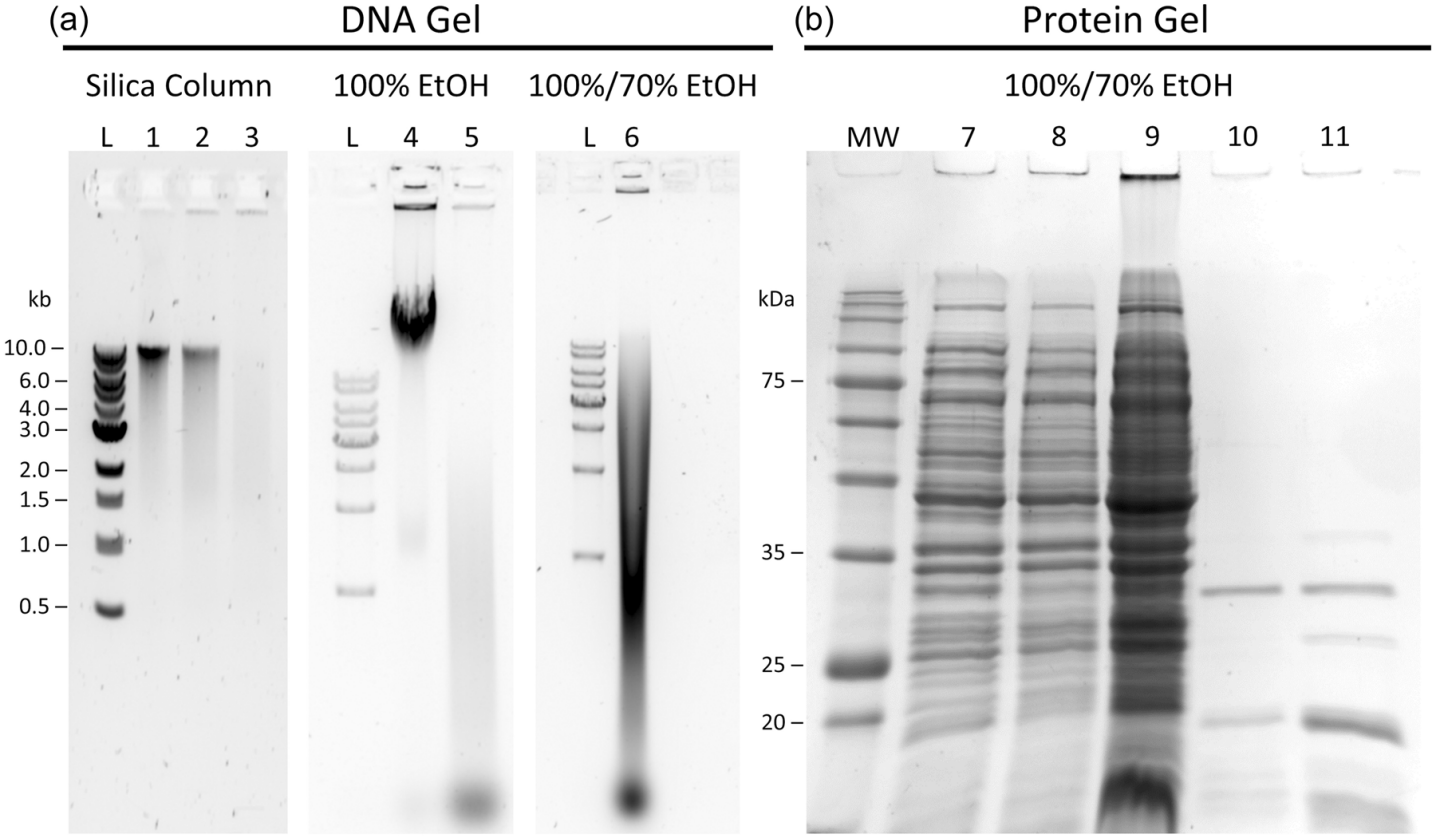
(a) Agarose gel analysis of extracted DNA confirms the presence of high molecular weight DNA consistent with the size of the *Escherichia coli* genome (4500–5500 kb). Subsequent wash steps using 70% EtOH and vigorous mixing lead to shearing of the DNA (lane 6). (1) gDNA purified by silica column. (2) gDNA purified by silica column and incubated with HindIII. (3) gDNA purified by silica column and incubated with HindIII and ExoIII. (4) gDNA purified by EtOH precipitation. (5) gDNA purified by EtOH precipitation and incubated with HindIII and ExoIII. (6) gDNA purified by 100% EtOH precipitation, followed by three 70% EtOH washes. L: 1 kb ladder. (b) SDS‐PAGE analysis showing the presence of protein contaminates in the purified gDNA. (7) *E. coli* JM110 cell pellet. Lane 8: *E. coli* JM110 pellet after incubation with lysis buffer (no Proteinase K, no RNaseA). Lane 9: gDNA purified from JM110 lysate without Proteinase K and Rnase, 100% EtOH precipitation, and 70% EtOH washes. Lane 10: *E. coli* JM110 pellet after incubation with lysis buffer, Proteinase K, and RnaseA. Lane 11: gDNA purified from JM110 lysate incubated with Proteinase K and RnaseA, 100% EtOH precipitation, and 70% EtOH washes. MW: NSR prestained protein molecular weight marker (mass in kDa).

A supply of genomic DNA was required as the starting material ([1] in Figure 1), for which bacterial DNA was selected because it is readily available, especially in any molecular biology lab. Bacterial DNA is also simple to purify, relative to isolating eukaryotic DNA which is stored in the nucleus and contains histones. *E. coli* JM110 was selected as the source of bacterial DNA because it lacks the adenosine and cytosine methyl transferases, which enables the production of nonmethylated dNTP products. Genomic bacterial DNA extracted with a modified protocol of the Monarch genomic DNA extraction kit (see previous section) gave the best yield. However, the silica columns normally used with the extraction kit give limited DNA binding capacity: yields of 148 and 183 ng/μL were obtained from two replicates of column purification, compared with 4080.0 ng/μL when the columns were replaced by ethanol precipitation. Agarose gel analysis confirms that both silica column purification and ethanol precipitation purification produce a high molecular weight band consistent with the size of the *E. coli* genome (Figure 2, lanes 1–4). Subsequent wash steps with 70% ethanol, in which the sample is subjected to vortexing, lead to shearing of the genomic DNA (Figure 2, lane 6), which can be minimized by reducing the amount of agitation. However, as the DNA will later be digested, DNA shearing is not a concern and seemed to have no effect on downstream processes.

After incubation of cell lysate with Proteinase K, there was a significant reduction in the amount of higher molecular weight protein contaminants, as observed by SDS‐PAGE analysis (Figure 2, lanes 10 and 11). The distinct band in lanes 10 and 11 corresponds to Proteinase K (28.9 kDa) and a fainter band around 13.7 kDa is consistent with RNaseA from bovine pancreas. The latter, together with other bands below 30 kDa are concentrated when the gDNA is ethanol precipitated and suspended in a smaller volume (Figure 2, lanes 9 and 11). Most of the endogenous *E. coli* proteins above 30 kDa (Figure 2, compare lanes 7–9 to lanes 10–11) were successfully removed during the extraction process, and the remaining protein is degraded and digested fragments below 30 kDa, which are expected to have lost native activity, and are not relevant to the following steps.

### 3.2 dNMP synthesis

**Figure 3:**
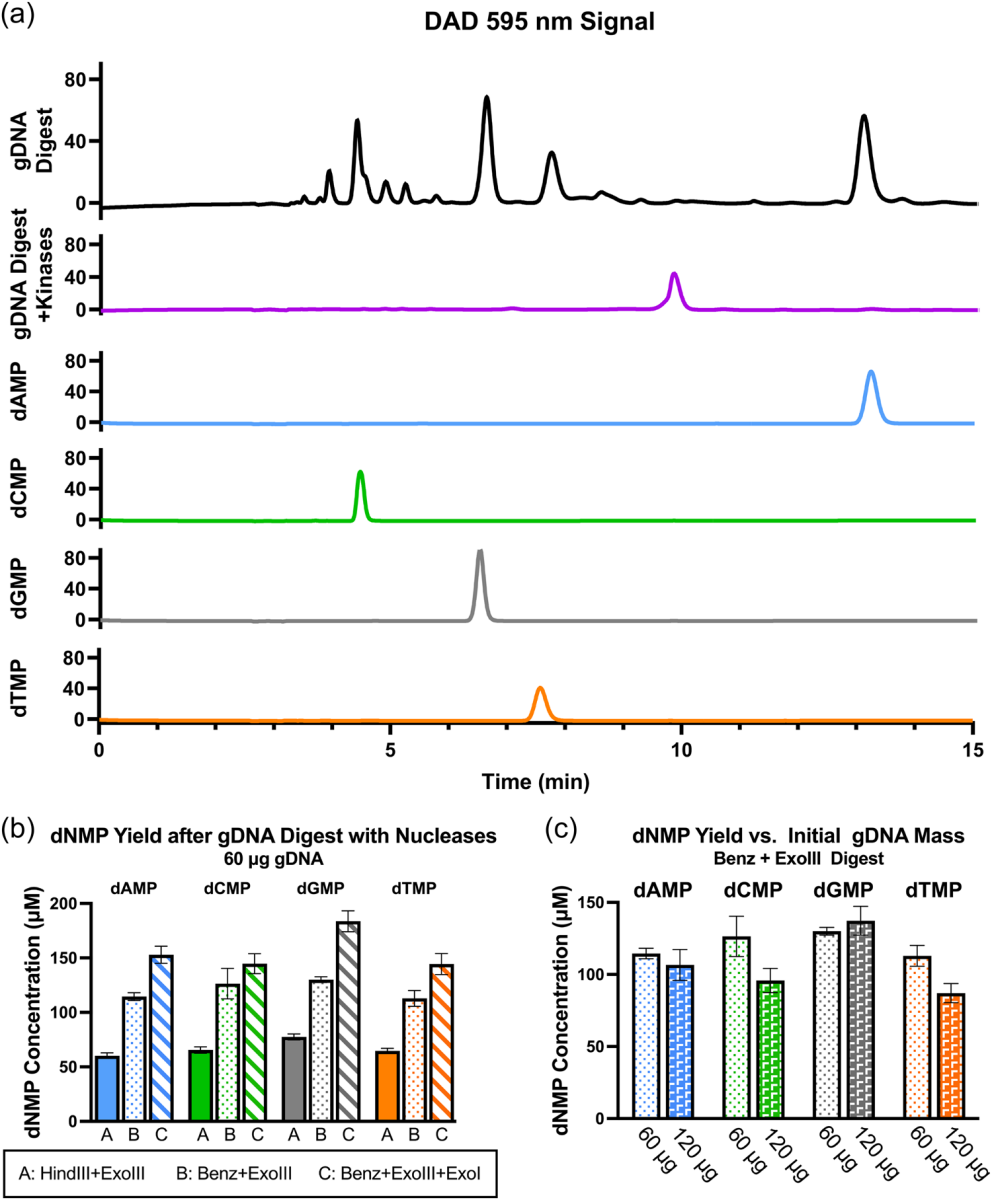
(a) Sample high pressure liquid chromatography (HPLC) signal curve for *t* = 0–15 min of 60 μg gDNA digest by 3.35 U Benz and 100 U ExoIII (black) and signal curves of 15 µM 2′‐deoxynucleotide 5′‐diphosphate (dNMP) standards. Peaks present in the gDNA digest correspond to the elution times of dNMP standards. After incubating the digestion sample with kinase enzymes (purple) the dNMP peaks are no longer visible, indicating successful conversion to 2′‐deoxynucleotide 5′‐triphosphate (dNTP) products. (line colors also correlate with plot for *t* = 15–40 min in Figure 6) (b) Concentration of dNMPs after incubation of gDNA with different digestion enzymes. Where listed: 3.35 U Benz, 40 U HindIII, 100 U ExoIII, or 20 U ExoI (c) Effect of gDNA mass on the yield of dNMPs. Varied mass of gDNA incubated with 3.35 U Benz and 100 U ExoIII. All samples were incubated with digestion enzymes for 16 h at 37°C. Data presented as the average and standard deviation (*n* = 3).

The precipitated gDNA was used for dNMP synthesis as shown in step 2, Figure 1. HindIII, an endonuclease that cuts at A/AGCTT sites, was used on the gDNA, leaving a 5′ overhang. ExoIII subsequently catalyzed the stepwise removal of dNMPs, initiating at the HindIII cut sites. Digestion of gDNA with HindIII and ExoIII was confirmed by Figure 2, lane 5 and showed successful production of dNMPs on HPLC (Figure 3a). The total average of all dNMP concentrations from a 60 μg digest of precipitated gDNA with HindIII and ExoIII was 268 µM (an average of 67 µM each dNTP). Assuming the average molecular weight of a basepair to be 660 g/mol, this corresponds 4.4 μg of digested DNA and an apparent 7% yield of dNMPs. The yield was approximately doubled to 13% by replacing HindIII with a nonspecific nuclease, Benzonase (Benz), which digests DNA into 3–5 bp long fragments (Figure 3b). The nonspecific nuclease also aids RNaseA in the digestion of contaminating RNAs. Increasing the initial mass of precipitated gDNA, does not increase the yield of dNMPs (Figure 3c), indicating that the rate is not diffusion controlled and the nuclease enzymes are operating above the *K*_M_.

Exonuclease III is reported to have activity only on dsDNA, producing ssDNA fragments and dNMPs. To improve yield, a single‐stranded nuclease, Exonuclease I, was added. This increased the dNMP yield to 17% (Figure 3b). It should be noted, however, that the accuracy with which yield is calculated is also influenced by the low accuracy in quantifying the initial mass of gDNA in the reaction, due to other contaminants absorbing near 260 nm. HPLC spectra also confirm that there are impurities absorbing at 259 nm. These could be small molecules naturally present in the *E. coli* cytoplasm and remnants of degraded RNA and proteins from the extraction process. Despite reducing the accuracy of yield determination (underestimating yield), the impurities were not shown to interfere with downstream applications of the dNTPs, and the dNMP peaks were still well resolved (Figure 3a).

Also of note is the variability in yield of different dNMPs (Table 1, Figure 3b,c). Exonuclease III is a processive enzyme that degrades stepwise in the 3′–5′ direction. Although the overall GC content of the *E. coli* genome is near 50% (Blattner et al., 1997), the amount of each dNMP that is produced is dependent on the GC content near the cut site of the HindIII or Benzonase enzyme. As benzonase is nonspecific, predicting the sequence of these cut‐sites is not possible. There may also be batch‐to‐batch variation in the quality of the extracted gDNA depending on variations in cell growth of the *E. coli* samples, which may affect the calculated yield. Although the cells were cultured under the same conditions for the same length of time, there is large variation in the measured optical density of the cells when harvested. Even when normalized to optical density measurements, there were still large differences in the final biomass of harvested cells (data not shown).

**Table 1:** Yield of in‐house expressed nucleotide kinase proteins.

Protein	Yield (mg/L)	Fold difference in specific activity after purification
adk	249 ± 44	1.51 ± 0.13
cmk	299 ± 43	1.91 ± 0.40
gmk	395 ± 153	2.36 ± 0.42
tmk	325 ± 60	2.53 ± 0.84
ndk	300 ± 197	1.60 ± 0.26

*Note*: Protein purity measured by fold difference in specific activity (measured in μmol/min/mg protein) of purified protein relative to *Escherichia coli* overexpression lysate. Data presented as the average and the standard error of the mean (*n* = 3).

Abbreviations: adk, adenylate kinase; cmk, cytidylate kinase; gmk, guanylate kinase; ndk, nucleotide disphosphate kinase; tmk, thymidylate kinase.

### 3.3 Overexpression of nucleotide kinases

The ability to produce dNTP products from deoxynucleoside or dNMP starting material with nucleotide kinases has been reported previously (step 3, Figure 1). Bao et al. demonstrated the use of dNMP kinases from yeast and pyruvate kinase from rabbit muscle to produce dNTP products (Bao & Ryu, 2007). Most recently, Loan et al. showed that *E. coli* cell‐free extract overexpressing recombinant *Drosophila melanogaster* nucleoside kinase can produce dNTPs at a concentration sufficient for PCR (Loan et al., 2019). The cell‐free extract was supplemented with commercial deoxynucleosides, and the *Drosophila* nucleoside kinase worked in conjunction with endogenous *E. coli* kinases to synthesize dNTPs. However, to our knowledge, the pathway to link this with dNMP production has not been reported with recombinant expression of the nucleoside kinases in *E. coli* together with integration of the enzyme generated dNMPs from gDNA for PCR.

**Figure 4:**
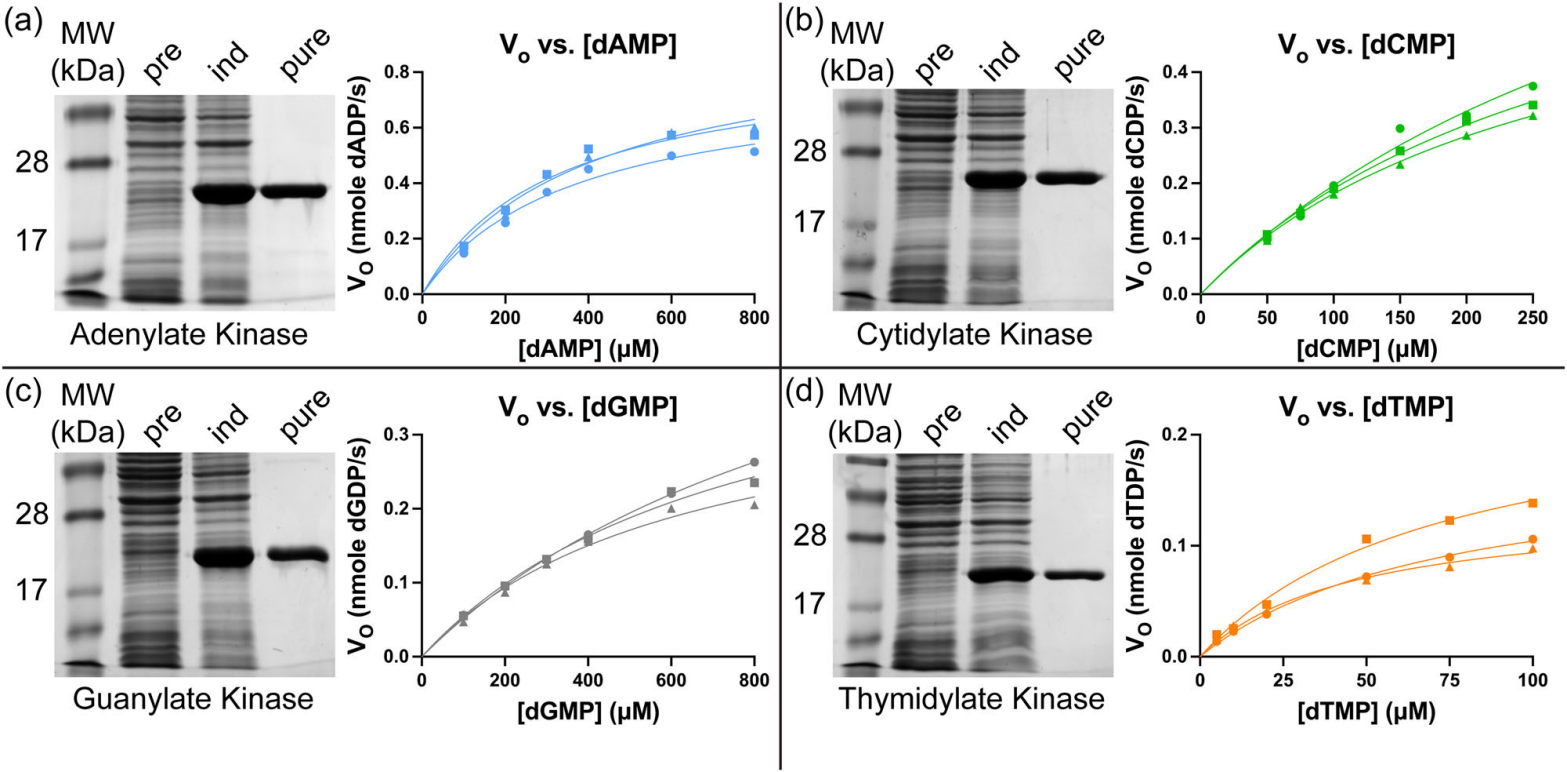
SDS‐PAGE analysis and Michaelis–Menten plots for (a) adenylate kinase, (b) cytidylate kinase, (c) guanylate kinase, and (d) thymidylate kinase. SDS‐PAGE analysis of molecular weight marker (MW) and BL21(DE3) cells overexpressing kinases before induction (pre), after induction (ind), and after purification with Ni‐NTA resin (pure). Michaelis–Menten plots show three independent replicates of kinetics performed on the same batch of each purified enzyme. MW: SeeBlue Plus2 prestained ladder.

**Figure 5:**
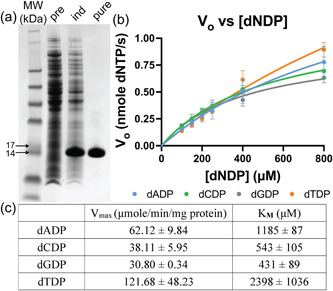
(a) SDS‐PAGE of BL21(DE3) cells overexpressing nucleotide diphosphate kinase before induction (pre), after induction (ind), and after purification with Ni‐NTA resin (pure). (b) Michaelis–Menten plot of purified NDK enzyme activity toward the four 2′‐deoxynucleotide 5′‐diphosphate substrates. (c) Kinetic parameters of in‐house produced nucleotide diphosphate kinase calculated by nonlinear regression with Michaelis–Menten equation.

Building on this previous work, overexpression and characterization of the kinases from *E. coli* were targeted: gene sequences from the *E. coli* DH5α genome for adk, cmk, gmk, tmk, and ndk were selected and assembled with a pET24a backbone including a C‐terminal histidine tag using Klenow assembly as described in the Methods section (Supporting Information: Tables S1 and S2). The native *E. coli* proteins were selected because they pose no problems with cell toxicity, codon optimization, or post‐translational modification, as we are using *E. coli* as the host cell expression system. Additionally, these enzymes have been extensively studied and information on their sequence and structure is widely available. Assembled plasmids were transformed into BL21(DE3) (an *E. coli* B strain) for expression. SDS‐PAGE gel analysis of pre‐induced and induced *E. coli* lysate confirms the overexpression of the desired proteins (Figures 4 and 5) and the Ni‐NTA purification. The purity of the proteins was assessed by measuring the specific activity in μmol/min/mg of total protein *E. coli* lysate, which overexpressed the kinases and then the specific activity of Ni‐NTA purified kinases. Negative control kinetics were taken from reactions of *E. coli* lysate (not overexpressing kinases) targeting dAMP, dCMP, dGMP, and dTMP which showed negligible activity of native kinases in the lysate for dCMP, dGMP, and dTMP. The negative control kinetic reaction of *E. coli* lysate against dADP did show some nonspecific native kinase activity. However, the overall purity as measured by the increase in specific activity following Ni‐NTA purification was of the same order of magnitude as purity determined by SDS‐PAGE image analysis (Supporting Information: Table S7).

Overall, there was a 1.51–2.53 fold improvement in activity of purified protein compared with protein in lysate. Although this enhancement is quite small, the protein gels appear to show impressive improvement in the protein purity from the lysate to purified sample (see the Coomassie‐stained bands, Figure 4). Nevertheless, the reaction velocities were lower than published values for these enzymes without a His tag which utilized a multi‐step chromatographic protocol to attain high purity (Table 2). This may contribute to the observed differences in the kinetic parameters, which were previously measured in a Tris‐HCl buffer, with MgCl_2_ (see Supporting Information: Table S3). In contrast, the anion used in this study was acetate, which can interfere with the kinase‐dNMP activity. Nevertheless, to link the kinase phosphorylation of dNMP to the gDNA digestion reaction and complete the pathway from gDNA to dNTP, the same buffer conditions were assessed here for both steps.

**Table 2:** Kinetic parameters of purified in‐house produced NMP kinases calculated by nonlinear regression with Michaelis–Menten equation.

	Reported values		Single batch in triplicate		Batch to batch comparison	
			*N* = 1		*N* = 3	
			*n* = 3		*n* = 1	
	*V*_max_ (μmole/min/mg protein)	*K*_M_ (μM)	*V*_max_ (μmole/min/mg protein)	*K*_M_ (μM)	V_max_ (μmole/min/mg protein)	*K*_M_ (μM)
Adenylate kinase	875 (Saint Girons et al., 1987)	850 (Saint Girons et al., 1987)	44.14 ± 4.06	359 ± 40	47.85 ± 10.64	359 ± 84
Cytidylate kinase	263 (Bucurenci et al., 1996)	94 (Bucurenci et al., 1996)	31.19 ± 7.18	334 ± 82	69.32 ± 31.80	314 ± 154
Guanylate kinase	187 (Oeschger, 1978)	300 (Oeschger, 1978)	42.95 ± 11.09	851 ± 270	76.16 ± 23.99	848 ± 402
Thymidylate kinase	50 (Munier‐Lehmann et al., 2001)	15 (Munier‐Lehmann et al., 2001)	16.87 ± 4.84	64 ± 16	39.41 ± 12.08	150 ± 22

*Note*: Variation shown within and between different batches of protein. Data presented as the average and standard deviation (*n* = 3).

Magnesium and potassium have also been shown to affect enzyme activity and affinity (Oeschger, 1978) although for the data reported here magnesium concentration was above the *K*_M_ (Supporting Information: Figure S2), and while higher than in previous reports, there was no evidence of reduced activity at this concentration (Supporting Information: Figure S2).

Also, consistent with the different buffer, the apparent *K*_M_ for all NMP kinases differed relative to published values (Table 2). The *K*_M_ of thymidylate kinase is much smaller than for other NMP kinases, suggesting high affinity of the enzyme for dTMP; this is consistent with previous findings. Although NMP kinases generally display high base specificity, adenylate, cytidylate, and guanylate kinase are reported to display some substrate promiscuity, although the activity is greatly reduced relative to the enzyme's preferred substrate. Cmk also phosphorylates UMP, and gmk has been shown to have some activity on adenine moieties. However, in this work, the activity of kinase enzymes toward their nonpreferred base was found to be minimal. This was shown by removal of a single kinase enzyme from the cascade, which resulted in failure to produce all four dNTPs (Supporting Information: Figure S17). Adk, cmk, and gmk act on both the ribose and deoxyribose forms of nucleosides, but generally, the kinases display higher affinity for the ribonucleotide. However, uracil monophosphate (UMP) (equivalent to the thymidine in RNA) is not a substrate of tmk. This explains why, in comparison to other NMP kinases, thymidylate kinase has the highest affinity for its deoxynucleoside substrate. In bacteria, phosphorylation of uracil is carried out by distinct UMP kinases, whereas in eukaryotes a single enzyme phosphorylates UMP and CMP (Bertrand et al., 2002). Additionally, the *V*_max_ of thymidylate kinase is slower than that of other NMP kinases, although the hypothesized phosphoryl transfer mechanism is the same (Brundiers et al., 1999). The lower V_max_ is probably the result of a mechanistical difference, such as slow product release, and therefore may also be associated with the increased affinity of thymidylate kinase for dTMP.

As expected, nucleotide diphosphate kinase (NDK) was active toward all four target dNDPs (Figure 5). The *V*_max_ values are of the same order of magnitude as the NMP kinases, but the *K*_M_s are much greater. The decreased affinity of the protein for specific nucleotide diphosphates is consistent with the promiscuity of this enzyme and the fact that it accepts all four bases.

### 3.4 dNTP synthesis and integration into PCR

**Figure 6:**
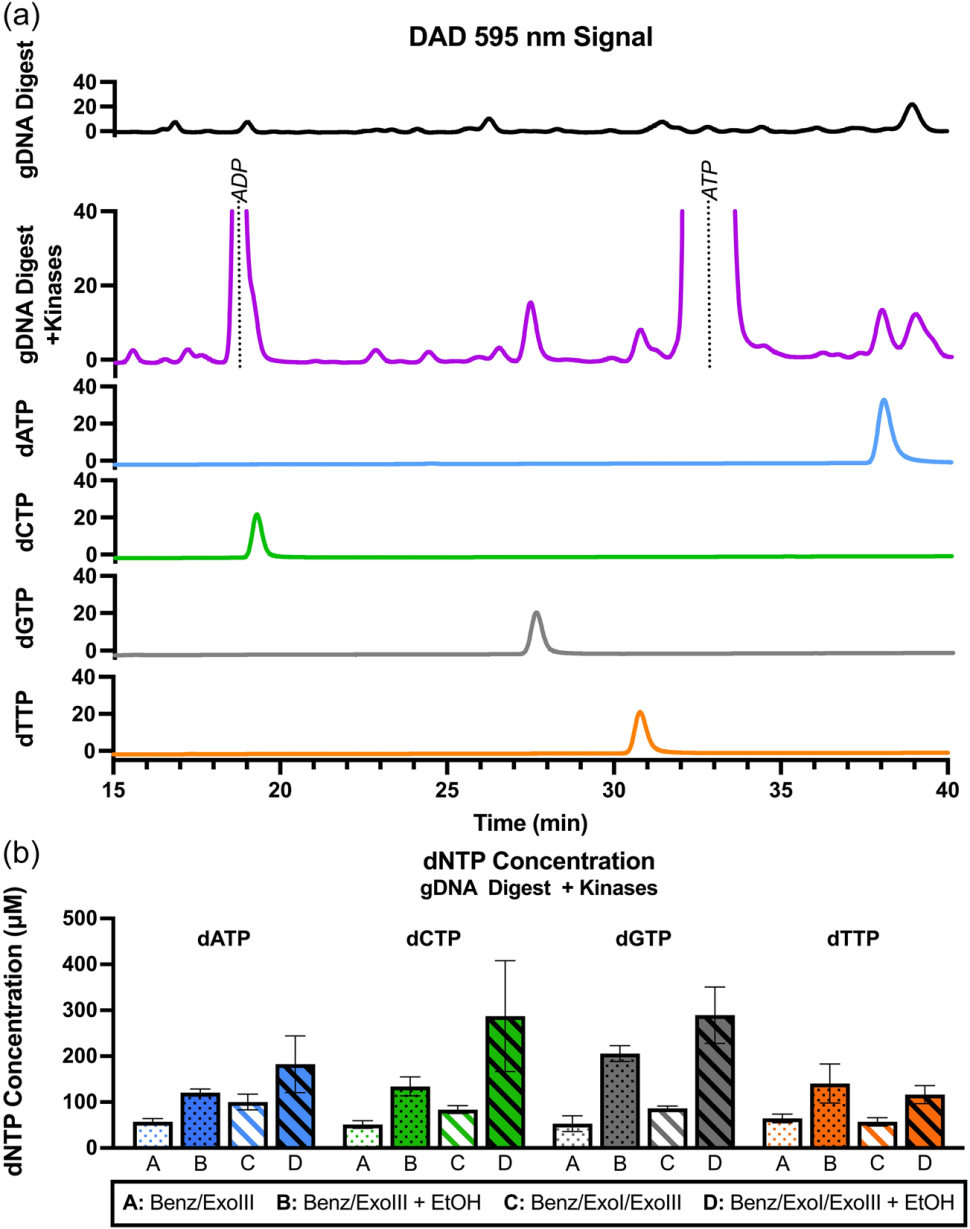
(a) Signal curve for high pressure liquid chromatography (HPLC) (*t* = 15–40 min) of 60 μg gDNA digest by Benz/ExoIII followed by incubation with kinase enzymes (purple) and signal curves of 15 μM 2′‐deoxynucleotide 5′‐triphosphate (dNTP) standards. Comparison of curve before incubation with kinase enzymes (black) confirms the presence of new peaks corresponding to the elution time of dNTP standards (see Figure 3 for *t* = 0–15 min). The peak at 38.18 min corresponds to the phosphoryl donor adenosine 5′‐triphosphate (ATP), present at high concentration to force the reaction in the forward direction. The peak at 18.69 min corresponds to adenosine 5′‐diphosphate (ADP) produced after phosphoryl transfer. ADP/dCTP peaks are not well resolved. (b) Concentration of in‐house dNTPs. Benz + ExoIII + ExoI: digestion of gDNA with benzonase, ExoIII and ExoI followed by incubation with kinases. Benz + ExoIII + EtOH: digestion of gDNA with benzonase and ExoIII followed incubation with kinases and EtOH precipitation.

The nucleotide kinase enzymes were integrated into the biocatalytic pathway (step 3, Figure 1) to produce dNTP products. dNTP molecules were identified on HPLC between *t* = 15–40 min, after incubation of gDNA digestion reactions with the kinases (Figure 6a, see Figure 3a for *t* = 0–15 min). In the sample after incubation with kinases (purple) the peaks corresponding to dNMPs are no longer present at earlier elution times (see Figure 3a), and dNTP peaks are present in Figure 6a, indicating successful conversion of dNMPs to dNTPs.

**Figure 7:**
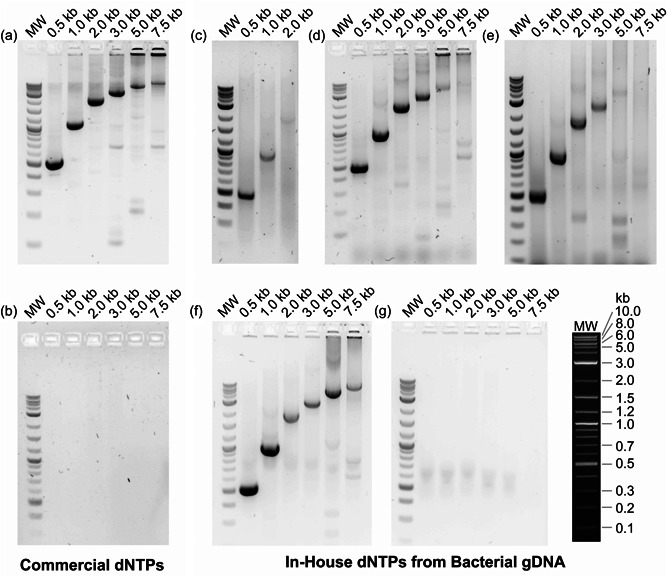
(a) Positive control lambda genome polymerase chain reaction (PCR) with commercial 2′‐deoxynucleotide 5′‐triphosphate (dNTPs). (b) Negative control lambda genome PCR (no dNTPs). (c) Lambda genome PCR with in‐house dNTPs from gDNA digest with HindIII/ExoIII (d) Lambda genome PCR with in‐house dNTPs from gDNA digest with Benz/ExoIII (e) Lambda genome PCR with in‐house dNTPs from gDNA digest with Benz/ExoI/ExoIII (f) Lambda genome PCR with in‐house dNTPs from gDNA digest with Benzonase/ExoIII and EtOH precipitation (g) Lambda genome PCR negative control with gDNA digest samples (no kinases).

A nucleic acid amplification protocol (step 4, Figure 1) using dNTPs synthesized from 60 μg gDNA digested with HindIII and ExoIII was capable of amplifying fragments up to 1 kb, but larger fragment PCRs failed (Figure 7c). When HindIII was replaced with Benzonase, samples were able to amplify fragments up to 5 kb, but the band brightness relative to the positive controls was still low (Figure 7d). PCR using dNTPs synthesized from Benzonase, ExoI, and ExoIII digestion, produced the same results as dNTPs synthesized from only Benzonase and ExoIII digestion, so the three‐enzyme system offers no advantage over the two‐enzyme system (Figure 7e).

The average yield of dNTPs generated from phosphorylation after digestion with the three‐enzyme system was 82 μM of each dNTP. These experiments reveal the limitation from the concentration of dNTPs, which is highly dependent on the starting concentration of dNMP substrate produced by gDNA digestion. Additionally, while the HPLC spectra demonstrates loss of dNMP peaks, there is only a 39%–66% conversion of dNMPs to the desired dNTP products (Table 3). The large ATP peak still present on the HPLC spectra implies that the concentration of the cosubstrate ATP is not limiting. However, the kinases are known to catalyze both the forward and reverse reactions. Within the reaction time, the system will reach an equilibrium between dNMP, dNDP, and dNTP concentrations, thus preventing complete substrate conversion.

**Table 3:** Yield of 2′‐deoxynucleotide 5′‐monophosphate (dNMP) and 2′‐deoxynucleotide 5′‐triphosphate (dNTP) products generated from a 60 μg gDNA digest with 3.35 U Benz, 20 U ExoI, and 100 U ExoIII and subsequent phosphorylation with in‐house produced kinases (no EtOH precipitation).

	Yield of dNTPs from Benz/ExoI/ExoIII digest and Kinase Incubation (μM)	Yield of dNMPs from Benz/ExoI/ExoIII digest (μM)	Percent conversion
A	100 ± 18	153 ± 8	66
C	84.8 ± 8.6	145 ± 9	58
G	86.5 ± 4.7	184 ± 10	47
T	57.0 ± 8.9	144 ± 10	39

*Note*: Percent conversion calculated as the concentration of dNTPs relative to the concentration of dNMPs. Data presented as the average and standard deviation (*n* = 3).

Attempts were made to increase the number of digestion enzymes; however, this also showed no increase in the yield of dNTPs (Supporting Information: Figure S21). This implies that although there is digestion of gDNA to small fragments that are not visible on agarose gel (Figure 7g), dNMPs are likely not the only product of the digestion reaction. The digestion reaction may produce nucleotides in other forms (such as deoxynucleosides) or leave short undigested fragments which cannot be converted into the desired dNTP products. To take the final step to achieve the dNTP concentration necessary for larger fragment PCR, the dNTPs were precipitated with ethanol and resuspended in a smaller volume to concentrate them (Supporting Information: Figure S18); The ethanol precipitation does not selectively precipitate dNTPs, but also likely precipitates other nucleotides and oligonucleotide fragments and salts.

The increased concentration of the dNTPs, as measured on HPLC (Figure 6b) enabled successful PCR amplification up to 7.5 kb and showed improved band brightness (Figure 7f). Applying ethanol precipitation increased the dNTP concentration in both the two‐enzyme and three‐enzyme system. The more economical two‐enzyme digestion system after phosphorylation gave an average yield of 150 μM for each dNTP. Negative controls were run supplementing the dNTP reaction solution with digested gDNA to confirm that the dNTPs were the product of the kinase reaction with the dNMPs, and not simply by‐products of the gDNA extraction and digestion (Figure 7g).

The yield, as determined by the fluorescence of Pico Green, in PCR reactions with in‐house dNTPs was comparable to a PCR reaction with 150 μM of commercial dNTPs (Supporting Information: Figure S19). Additionally, while the in‐house dNTPs do show false positives against the *E. coli* genome due to the presence of undigested *E. coli* genome fragments (Supporting Information: Figure S20), there appears to be little interference from other contaminants with the PCR reactions. The nonspecific banding pattern observed in the in‐house dNTP PCR samples is strikingly similar to the nonspecific banding pattern in the commercial dNTP samples (Figure 7). The presence of salts from the dNTP reaction buffer (which may also co‐precipitate with dNTPs during EtOH precipitation), may also affect polymerase activity, especially for polymerase enzymes which are sensitive to high concentrations of magnesium. However, the polymerase used in this study (DeepVent) tolerates the high salt concentration, and is also identical to the open‐access version, OpenVent, which has been successfully produced and utilized in low‐resource contexts (Bhadra et al., 2021). The method for producing in‐house dNTPs presented in this study and the previously published open‐source OpenVent polymerase are compatible with one another and together will enhance accessibility to nucleic acid amplification in frugal labs.

## 4 CONCLUSION

A first proof of concept to provide a reagent pathway from genomic DNA to PCR is reported, whereby dNTPs were synthesized from bacterial genomic DNA and the endogenous nucleotide kinase enzymes of *E. coli* were successfully overexpressed, purified, and subsequently found to be active toward target substrates. gDNA extracted from *E. coli* JM110 produced dNMPs when incubated with DNA digestion enzymes in ~15% yield. Given that there is an easy source of *E. coli* genomic DNA, higher yield is not necessarily required and with the ethanol precipitation method, a precipitated dNMP product was shown to be adequate for PCR use. The dNMP products incubated with kinase enzymes and ATP, followed by EtOH precipitation, yielded dNTPs at concentrations of 150 μM each dNTP, sufficient for performing PCR up to 7.5 kb.

As the aim of our work is to promote an integrated route from gDNA to PCR and reduce supply chain dependency in research laboratories without access to reagent kits for NAATs, this is the first step to prove the principle of in‐house production of dNTPs. The results have revealed the importance of identifying an optimized buffer solution for use across the digestion and phosphorylation steps to increase dNTP yields and design of a simple lysis buffer will also remove reliance on the proprietary solution.

There is also potential for better yield, performance, and reusability through improved isolation and immobilization techniques, designed through protein engineering that we have reported previously (Henderson et al., 2019; Seevaratnam et al., 2022). The next step is the enzyme engineering and auto immobilization on silica of the key kinase enzymes, so that the workflow can be designed into reusable dNTP producing reaction columns.

To further reduce supply chain dependency, this will form a foundation to add (a) an ATP regeneration system (b) design and express a DNA digestion enzyme in‐house and (c) optimize the gDNA extraction protocol. ATP regeneration systems have been successfully implemented in biocatalytic synthesis by Fehlau et al. (2020) and Alissandratos et al. (2016), but since digestion enzymes attack host DNA, leading to cell death recombinant expression of enzymes for this step requires modified techniques, such as cell‐free or periplasmic expression. Nevertheless, overall, this synthesis method shows great potential to be further developed into an accessible method to produce dNTPs in low‐resource settings.

## Supporting information

Supporting information.

## Data Availability

All raw data files are available on the University of Cambridge institutional repository (Apollo).

## References

[bit28374-bib-0001] Africa Renewal . (2020). *Public financing for health in Africa: 15% of an elephant is not 15% of a chicken*. https://www.un.org/africarenewal/magazine/october-2020/public-financing-health-africa-when-15-elephant-not-15-chicken

[bit28374-bib-0002] Agarwal, R. P. , Robison, B. , & Parks, R. E. (1978). Nucleoside diphosphokinase from human erythrocytes. Methods in Enzymology, 51(C), 376–386. 10.1016/S0076-6879(78)51051-3 211386

[bit28374-bib-0003] Ahmad, A. L. , Oh, P. C. , & Abd Shukor, S. R. (2009). Sustainable biocatalytic synthesis of L‐homophenylalanine as pharmaceutical drug precursor. Biotechnology Advances, 27, 286–296. 10.1016/j.biotechadv.2009.01.003 19500550

[bit28374-bib-0004] Alissandratos, A. , Caron, K. , Loan, T. D. , Hennessy, J. E. , & Easton, C. J. (2016). ATP recycling with cell lysate for enzyme‐catalyzed chemical synthesis, protein expression and PCR. ACS Chemical Biology, 11(12), 3289–3293. 10.1021/acschembio.6b00838 27978706

[bit28374-bib-0005] Bao, J. , & Ryu, D. D. Y. (2007). Total biosynthesis of deoxynucleoside triphosphates using deoxynucleoside monophosphate kinases for PCR application. Biotechnology and Bioengineering, 98(1), 1–11. 10.1002/bit.21498 17514761

[bit28374-bib-0006] Bertrand, T. , Briozzo, P. , Assairi, L. , Ofiteru, A. , Bucurenci, N. , Munier‐Lehmann, H. , Golinelli‐Pimpaneau, B. , Bârzu, O. , & Gilles, A. M. (2002). Sugar specificity of bacterial CMP kinases as revealed by crystal structures and mutagenesis of *Escherichia coli* enzyme. Journal of Molecular Biology, 315(5), 1099–1110. 10.1006/jmbi.2001.5286 11827479

[bit28374-bib-0007] Bhadra, S. , Nguyen, V. , Torres, J. A. , Kar, S. , Fadanka, S. , Gandini, C. , Akligoh, H. , Paik, I. , Maranhao, A. C. , Molloy, J. , & Ellington, A. D. (2021). Producing molecular biology reagents without purification. PLoS One, 16(6), e0252507. 10.1371/JOURNAL.PONE.0252507 34061896 PMC8168896

[bit28374-bib-0008] Blattner, F. R. , Plunkett, G. , Bloch, C. A. , Perna, N. T. , Burland, V. , Riley, M. , Collado‐Vides, J. , Glasner, J. D. , Rode, C. K. , Mayhew, G. F. , Gregor, J. , Davis, N. W. , Kirkpatrick, H. A. , Goeden, M. A. , Rose, D. J. , Mau, B. , & Shao, Y. (1997). The complete genome sequence of *Escherichia coli* K‐12. Science, 277(5331), 1453–1462. 10.1126/SCIENCE.277.5331.1453/ASSET/5AE101DE-877C-44DD-B176-3A8D1DA63BFE/ASSETS/GRAPHIC/SE3275565002.JPEG 9278503

[bit28374-bib-0009] Brundiers, R. , Lavie, A. , Veit, T. , Reinstein, J. , Schlichting, I. , Ostermann, N. , Goody, R. S. , & Konrad, M. (1999). Modifying human thymidylate kinase to potentiate azidothymidine activation. Journal of Biological Chemistry, 274(50), 35289–35292. 10.1074/jbc.274.50.35289 10585390

[bit28374-bib-0010] Bucurenci, N. , Sakamoto, H. , Briozzo, P. , Palibroda, N. , Serina, L. , Sarfati, R. S. , Labesse, G. , Briand, G. , Danchin, A. , Bârzu, O. , & Gilles, A. M. (1996). CMP kinase from *Escherichia coli* is structurally related to other nucleoside monophosphate kinases. Journal of Biological Chemistry, 271(5), 2856–2862. 10.1074/jbc.271.5.2856 8576266

[bit28374-bib-0011] Burgess, K. , & Cook, D. (2000). Syntheses of nucleoside triphosphates. Chemical Reviews, 100(6), 2047–2060. 10.1021/cr990045m 11749283

[bit28374-bib-0012] Caton‐Williams, J. , Smith, M. , Carrasco, N. , & Huang, Z. (2011). Protection‐free one‐pot synthesis of 2′‐deoxynucleoside 5′‐triphosphates and DNA polymerization. Organic Letters, 13(16), 4156–4159. 10.1021/OL201073E 21790120 PMC3163101

[bit28374-bib-0013] Engel, N. , Wachter, K. , Pai, M. , Gallarda, J. , Boehme, C. , Celentano, I. , & Weintraub, R. (2016). Addressing the challenges of diagnostics demand and supply: Insights from an online global health discussion platform. BMJ Global Health, 1(4), e000132. 10.1136/BMJGH-2016-000132 PMC5321377 28588980

[bit28374-bib-0014] Fehlau, M. , Kaspar, F. , Hellendahl, K. F. , Schollmeyer, J. , Neubauer, P. , & Wagner, A. (2020). Modular enzymatic cascade synthesis of nucleotides using a (d)ATP regeneration system. Frontiers in Bioengineering and Biotechnology, 8, 854. 10.3389/fbioe.2020.00854 32903716 PMC7438870

[bit28374-bib-0015] Gao, C. , Wong, J. J. Y. , & Hall, E. A. H. (2023). TbEu(BTC) as a dNTP label for LAMP outcome in nucleic acid testing. Electroanalysis, e202200504. 10.1002/elan.202200504

[bit28374-bib-0016] Gibbs, R. A. (1990). DNA amplification by the polymerase chain reaction. Analytical Chemistry, 62(13), 1202–1214. 10.1021/ac00212a004 2196835

[bit28374-bib-0018] Hanahan, D. , Jessee, J. , & Bloom, F. R. (1991). Plasmid transformation of *Escherichia coli* and other bacteria. Methods in Enzymology, 204(C), 63–113. 10.1016/0076-6879(91)04006-A 1943786

[bit28374-bib-0019] Haynie, S. L. , & Whitesides, G. M. (1990). Preparation of a mixture of nucleoside triphosphates suitable for use in synthesis of nucleotide phosphate sugars from ribonucleic acid using nuclease P1, a mixture of nucleoside monophosphokinases and acetate kinase. Applied Biochemistry and Biotechnology, 23, 205–220. 10.1007/BF02942055 1693485

[bit28374-bib-0020] Henderson, C. J. , Pumford, E. , Seevaratnam, D. J. , Daly, R. , & Hall, E. A. H. (2019). Gene to diagnostic: Self immobilizing protein for silica microparticle biosensor, modelled with sarcosine oxidase. Biomaterials, 193, 58–70. 10.1016/J.BIOMATERIALS.2018.12.003 30562636

[bit28374-bib-0021] Le, T. , Ashrafi, E. H. , & Paul, N. (2009). Enhancing multiplex PCR efficiency using hot start DNTPs. Biotechniques, 47(5), 972–973. 10.2144/000113298

[bit28374-bib-0022] Loan, T. D. , Easton, C. J. , & Alissandratos, A. (2019). DNA amplification with in situ nucleoside to DNTP synthesis, using a single recombinant cell lysate of *E. coli* . Scientific Reports, 9(1), 15621. 10.1038/s41598-019-51917-z 31666578 PMC6821818

[bit28374-bib-0023] Lorenz, T. C. (2012). Polymerase chain reaction: Basic protocol plus troubleshooting and optimization strategies. Journal of Visualized Experiments, 63(63), 3998. 10.3791/3998 PMC4846334 22664923

[bit28374-bib-0024] Lovett, S. T. (2011). The DNA exonucleases of *Escherichia coli* . EcoSal Plus, 4(2), 1–30. 10.1128/ecosalplus.4.4.7 PMC4238392 26442508

[bit28374-bib-0025] MacKintosh, M. , Mugwagwa, J. , Banda, G. , Tibandebage, P. , Tunguhole, J. , Wangwe, S. , & Karimi Njeru, M. (2018). Health‐industry linkages for local health: Reframing policies for African health system strengthening. Health Policy and Planning, 33(4), 602–610. 10.1093/HEAPOL/CZY022 29562286 PMC5894083

[bit28374-bib-0026] Mahony, J. B. , Blackhouse, G. , Babwah, J. , Smieja, M. , Buracond, S. , Chong, S. , Ciccotelli, W. , O'Shea, T. , Alnakhli, D. , Griffiths‐Turner, M. , & Goeree, R. (2009). Cost analysis of multiplex PCR testing for diagnosing respiratory virus infections. Journal of Clinical Microbiology, 47(9), 2812–2817. 10.1128/JCM.00556-09 19571025 PMC2738055

[bit28374-bib-0027] Markoulatos, P. , Siafakas, N. , & Moncany, M. (2002). Multiplex polymerase chain reaction: A practical approach. Journal of Clinical Laboratory Analysis, 16(1), 47–51. 10.1002/JCLA.2058 11835531 PMC6808141

[bit28374-bib-0028] Matute, T. , Nuñez, I. , Rivera, M. , Reyes, J. , Blázquez‐Sánchez, P. , Arce, A. , Brown, A. J. , Gandini, C. , Molloy, J. , Ramirez‐Sarmiento, C. A. , & Federici, F. (2021). Homebrew reagents for low cost RT‐LAMP. *medRxiv: The Preprint Server for Health Sciences*. 10.1101/2021.05.08.21256891 PMC8730520 35027869

[bit28374-bib-0029] Mote, R. D. , Laxmikant, V. S. , Singh, S. B. , Tiwari, M. , Singh, H. , Srivastava, J. , Tripathi, V. , Seshadri, V. , Majumdar, A. , & Subramanyam, D. (2021). A cost‐effective and efficient approach for generating and assembling reagents for conducting real‐time PCR. Journal of Biosciences, 46(4), 109. 10.1007/S12038-021-00231-W/FIGURES/4 34845993 PMC8626763

[bit28374-bib-0030] Munier‐Lehmann, H. , Chaffotte, A. , Pochet, S. , & Labesse, G. (2001). Thymidylate kinase of mycobacterium tuberculosis: A chimera sharing properties common to eukaryotic and bacterial enzymes. Protein Science, 10(6), 1195–1205. 10.1110/ps.45701 11369858 PMC2374024

[bit28374-bib-0031] Oeschger, M. P. (1978). Guanylate kinase from *Escherichia coli* B. Methods in Enzymology, 51(C), 473–482. 10.1016/S0076-6879(78)51065-3 211389

[bit28374-bib-0032] OpenWetWare . *Klenow assembly method: Seamless cloning*. https://openwetware.org/wiki/Klenow_Assembly_Method:_Seamless_cloning

[bit28374-bib-0033] OpenWetWare . *Lidstrom: Autoinduction media*. https://openwetware.org/wiki/Lidstrom:Autoinduction_Media

[bit28374-bib-0034] OpenWetWare . *TOP10 chemically competent cells*. https://openwetware.org/wiki/TOP10_chemically_competent_cells#CCMB80_buffer

[bit28374-bib-0035] Reischl, A. T. , Schreiner, D. , Poplawska, K. , Kidszun, A. , Zepp, F. , Gröndahl, B. , & Gehring, S. (2020). The clinical impact of PCR‐based point‐of‐care diagnostic in respiratory tract infections in children. Journal of Clinical Laboratory Analysis, 34(5), e23203. 10.1002/JCLA.23203 32032458 PMC7228252

[bit28374-bib-0036] Rowley, S. , Garcia‐Gonzalez, P. , Radich, J. P. , Novakowski, A. K. , Usherenko, I. , & Babigumira, J. B. (2021). Analysis of the gap in PCR monitoring availability for patients with chronic myeloid leukemia in 60 low‐ and middle‐income countries. Cost Effectiveness and Resource Allocation, 19(1), 18. 10.1186/S12962-021-00271-X/FIGURES/1 33712039 PMC7953726

[bit28374-bib-0037] Roy, B. , Depaix, A. , Périgaud, C. , & Peyrottes, S. (2016). Recent trends in nucleotide synthesis. Chemical Reviews, 116, 7854–7897. 10.1021/acs.chemrev.6b00174 27319940

[bit28374-bib-0017] Saint Girons, I. , Gilles, A. M. , Margarita, D. , Michelson, S. , Monnot, M. , Fermandjian, S. , Danchin, A. , & Barzu, O. (1987). Structural and catalytic characteristics of *Escherichia coli* adenylate kinase. Journal of Biologial Chemistry, 262(2), 622–629. 10.1016/S0021-9258(19)75829-3 3027060

[bit28374-bib-0038] Seevaratnam, D. , Ansah, F. , Aniweh, Y. , Awandare, G. A. , & Hall, E. A. H. (2022). Analysis and validation of silica‐immobilised BST polymerase in loop‐mediated isothermal amplification (LAMP) for malaria diagnosis. Analytical and Bioanalytical Chemistry, 414(21), 6309–6326. 10.1007/S00216-022-04131-2 35657389 PMC9163865

[bit28374-bib-0039] Simarro, J. , Pérez‐Simó, G. , Mancheño, N. , Ansotegui, E. , Muñoz‐Núñez, C. F. , Gómez‐Codina, J. , Juan, Ó. , & Palanca, S. (2022). Technical validation and clinical implications of ultrasensitive PCR approaches for EGFR‐Thr790Met mutation detection in pretreatment FFPE samples and in liquid biopsies from non‐small cell lung cancer patients. International Journal of Molecular Sciences, 23(15), 8526. 10.3390/IJMS23158526 35955661 PMC9369170

[bit28374-bib-0040] Taegtmeyer, M. , Beeching, N. J. , Scott, J. , Seddon, K. , Jamieson, S. , Squire, S. B. , Mwandumba, H. C. , Miller, A. R. O. , Davies, P. D. O. , & Parry, C. M. (2008). The clinical impact of nucleic acid amplification tests on the diagnosis and management of tuberculosis in a British hospital. Thorax, 63(4), 317–321. 10.1136/THX.2007.083816 18024540

[bit28374-bib-0041] Wiersinga, W. J. , Hovius, J. , Windt, G. , Meijers, J. , Roelofs, J. , Dondorp, A. , Levi, M. , Day, N. , Peacock, S. , & Poll, T. (2009). Urokinase receptor is necessary for bacterial defense against gram‐negative sepsis (melioidosis) by facilitating phagoctytosis. Critical Care, 13(4), P1. 10.1186/CC8065

[bit28374-bib-0042] Wilson, K. R. , Kohler, J. C. , & Ovtcharenko, N. (2012). The make or buy debate: Considering the limitations of domestic production in Tanzania. Globalization and health, 8(1), 20. 10.1186/1744-8603-8-20/METRICS 22747578 PMC3413540

[bit28374-bib-0043] World Health Organisation . (2019). *Interagency statement on promoting local production of medicines and other health technologies*. https://www.who.int/publications/m/item/interagency-statement-on-promoting-local-production-of-medicines-and-other-health-technologies

[bit28374-bib-0044] Yanisch‐Perron, C. , Vieira, J. , & Messing, J. (1985). Improved M13 phage cloning vectors and host strains: Nucleotide sequences of the M13mpl8 and PUC19 vectors. Gene, 33(1), 103–119. 10.1016/0378-1119(85)90120-9 2985470

[bit28374-bib-0045] Yin, J. , Chen, S. , Zhang, N. , & Wang, H. (2018). Multienzyme cascade bioreactor for a 10 min digestion of genomic DNA into single nucleosides and quantitative detection of structural DNA modifications in cellular genomic DNA. ACS Applied Materials & Interfaces, 10(26), 21883–21890. 10.1021/acsami.8b05399 29882639

[bit28374-bib-0046] Zhang, H. L. , Omondi, M. W. , Musyoka, A. M. , Afwamba, I. A. , Swai, R. P. , Karia, F. P. , Muiruri, C. , Reddy, E. A. , Crump, J. A. , & Rubach, M. P. (2016). Challenges of maintaining good clinical laboratory practices in low‐resource settings. American Journal of Clinical Pathology, 146(2), 199–206. 10.1093/ajcp/aqw083 27473737 PMC6410885

[bit28374-bib-0047] Zou, H. , Cai, G. , Cai, W. , Li, H. , Gu, Y. , Park, Y. , & Meng, F. (2008). Extraction and DNA digestion of 5′‐Phosphodiesterase from malt root. Tsinghua Science and Technology, 13(4), 480–484. 10.1016/S1007-0214(08)70077-4

