# Supplementary material for: Biocatalytic synthesis of 2′‐deoxynucleotide 5′‐triphosphates from bacterial genomic DNA: Proof of principle
Source: Biotechnol Bioeng. 2023 Mar 25;120(6):1531–44. doi: 10.1002/bit.28374 (PMC10952841; doi:10.1002/bit.28374)
Supplement: Supplementary file 1 — Supporting information. [file BIT-120-1531-s001.pdf]

Supplemental Data

Contents

Plasmid Maps	2
Nucleotide Kinase DNA Sequences	3
Enzyme Kinetics Dependence on Magnesium Concentration	5
Buffer Composition of Enzyme Kinetics Reactions	6
Protein Extinction Coefficients	7
Lineweaver Burk Plots – NMP Kinases	8
NMP Kinases – Batch to Batch Variation	9
Kinetic Parameter Comparison	10
Lineweaver Burk Plots – Nucleotide Disphosphate Kinase	11
Nucleotide Diphosphate Kinase – Batch to Batch Comparison	12
Kinetics of Unpurified Kinase Lysate	13
Negative Control BL21(DE3) Lysate Kinetics	15
SDS-PAGE Image Analysis	17
HPLC dNMP Standard Curves	18
HPLC dNTP Standard Curves	19
HPLC ADP and ATP Elution Time	20
Lambda DNA PCR	22
Kinase Knockout	22
Effect of EtOH Precipitation on Synthesized dNTPs	23
PCR Yield	24
E. coli genome PCR	25
Increased Digestion Enzyme Concentration	26
dNTP Longevity	27
References	28

Plasmid Maps

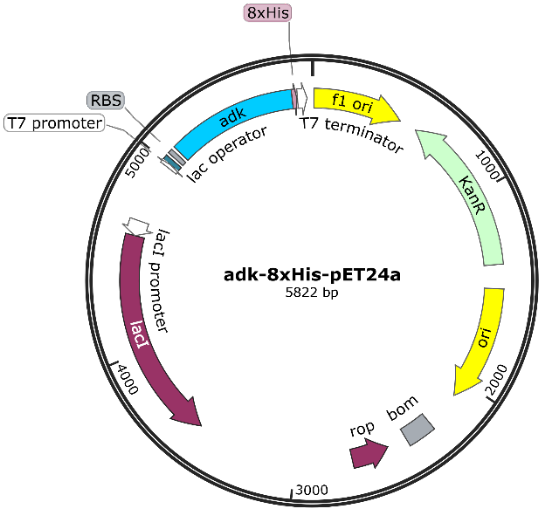

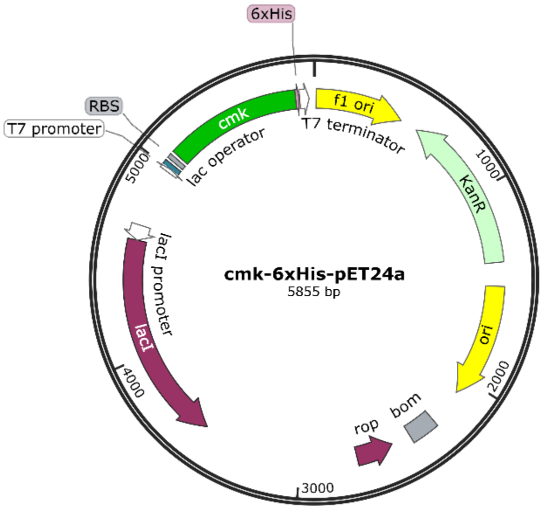

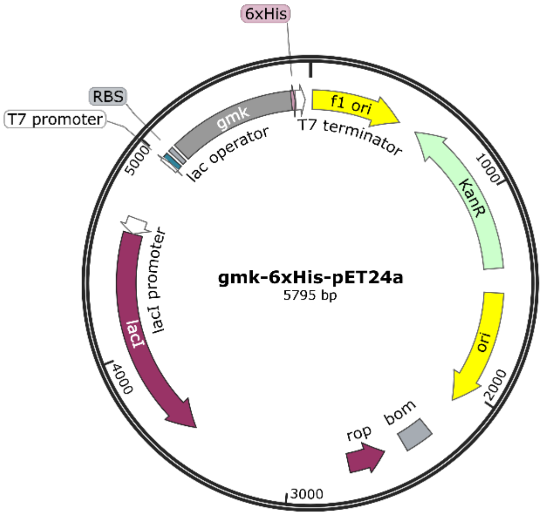

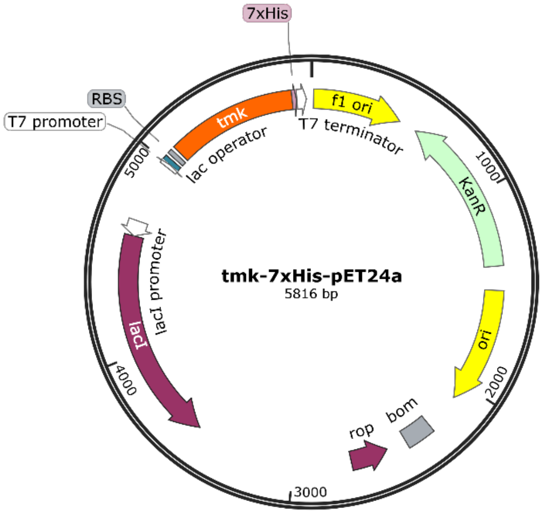

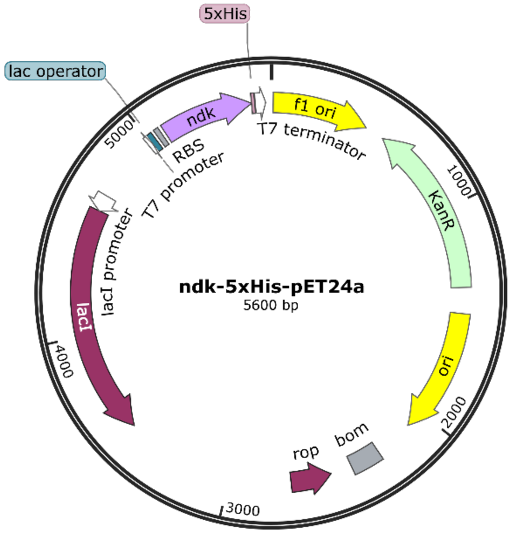

Figure S1. Plasmid maps of nucleotide kinases

Nucleotide Kinase DNA Sequences

Table S1. NCBI accession numbers for NMP kinase and NDK genes from *E. coli* DH5α

	dAMP Kinase	dCMP Kinase	dGMP Kinase	dTMP Kinase	Nucleotide Diphosphate Kinase
NCBI Accession Number	X03038	U00096.3	M84400	U41456.1	X57555
Protein MW including His tag (kDa)	24.7	25.6	24.4	24.7	16.1

Table S2. Primers for amplifying kinase genes from *E. coli* DH5α genome

Primers	Forward primer	Reverse primer
adk	ATGCGTATCATTCTGCTTGG	TCAGTGGTGGTGGTGGTGGTGGCCGAGGATTTTTTCCAGAT
cmk	GAGATATACATATGGGATCCATGACGGCAATTGCCCC	TCAGTGGTGGTGGTGGTGGTGTGCGAGAGCCAATTTCTG
gmk	GAGATATACATATGGGATCCATGGCTCAAGGCACGCTTTA	TCAGTGGTGGTGGTGGTGGTGGTCTGCCAACAATTTGCTGA
tmk	GAGATATACATATGGGATCCATGCGCAGTAAGTATATCGTCATTG	TCAGTGGTGGTGGTGGTGGTGTGCGTCCAACTCCTTCAC
ndk	ATACATATGGGATCCATGGCTATTGAACGTACTTTTTCCATCATCAAACC	GTGGTGGTGGTGGTGACGGGTGCGCGGGCAC

DNA sequencing results of protein encoding region of assembled nucleotide kinase plasmids. Red letters indicate point mutations that differ from the NCBI sequence, but do not alter the protein sequence.

adk-*8xHis*

ATGCGTATCATTCTGCTTGGCGCTCCGGGCGCGGGGAAAGGGACTCAGGCTCAGTTCATCATGGAGAA
ATATGGTATTCCGCAAATCTCCACTGGCGATATGCTGCGTGCTGCGGTCAAATCTGGCTCCGAGCTGG
GTAAACAAGCAAAAGACATTATGGATGCTGGCAAACTGGTCACCGACGAACTGGTGATCGCGCTGGTT
AAAGAGCGCATTGCTCAGGAAGACTGCCGTAATGGTTTCCTGTTGGACGGCTTCCCGCGTACCATTCC
GCAGGCAGACGCGATGAAAGAAGCGGGCATCAATGTTGATTACGTTCTGGAATTCGACGTACCGGAC
GAACTGATCGTTGACCGTATCGTCGGTCGCCGCGTTCATGCGCCGTCTGGTCGTGTTTATCACGTTAAA
TTCAATCCGCCGAAAGTAGAAGGCAAAGACGACGTTACCGGTGAAGAACTGACTACCCGTAAAGATG
ATCAGGAAGAGACCGTACGTAAACGTCTGGTTGAATACCATCAGATGACAGCACCGCTGATCGGCTAC
TACTCCAAAGAAGCAGAAGCGGGTAATACCAAATACGCGAAAGTTGACGGCACCAAGCCGGTTGCTG
AAGTTCGCGCTGATCTGGAAAAAATCCTCGGC*CACCACCACCACCACCACCACCAC*TGA

cmk-*6xHis*

ATGACGGCAATTGCCCCGGTTATTACCATTGATGGCCCAAGCGGTGCAGGGAAAGGCACCTTGTGTAA
GGCTATGGCGGAAGCGTTGCAATGGCATCTGCTGGACTCGGGTGCAATTTATCGCGTACTGGCATTGG
CGGCATTACATCACCATGTTGATGTTGCGTCGGAAGATGCGCTGGTACCGCTGGCATCCCATCTGGAT
GTACGTTTTGTGTCGACCAATGGCAATCTGGAAGTGATCCTCGAAGGGGAAGATGTCAGCGGCGAAAT
TCGTACTCAGGAAGTGGCGAATGCAGCTTCACAAGTCGCGGCATTCCCACGCGTTCGTGAAGCATTAT
TGCGTCGCCAACGCGCGTTTCGCGAATTACCAGGTCTGATTGCCGATGGCCGCGACATGGGAACGGTG

GTATTCCCTGATGCACCAGTGAAAATTTTCCTTGACGCCTCCTCGGAAGAACGTGCGCATCGCCGCATG
CTACAGTTGCAGGAGAAGGGCTTTAGTGTTAACTTTGAGCGCCTTTTGGCCGAGATCAAAGAACGCGA
CGACCGCGATCGTAACCGAGCGGTAGCGCCACTGGTTCCGGCAGCCGATGCTTTAGTGTTGGATTCCA
CCACCTTAAGCATTGAGCAAGTGATTGAAAAAGCGCTACAATACGCGCGCCAGAAATTGGCTCTCGCA
*CACCACCACCACCACCAC*TGA

gmk-*6xHis*

ATGGCTCAAGGCACGCTTTATATTGTTTCTGCCCCCAGTGGCGCGGGTAAATCCAGCCTGATTCAGGCT
TTATTAAAAACCCAACCGTTGTATGACACCCAGGTTTCTGTTTCACACACCACACGCCAACCGCGTCCT
GGTGAAGTCCACGGTGAACATTATTTCTTTGTTAATCATGATGAATTTAAAGAAATGATTAGCAGAGA
TGCGTTCCTCGAACACGCAGAAGTTTTTGGTAATTACTATGGCACTTCGCGTGAGGCCATTGAGCAAGT
ACTGGCGACCGGTGTCGATGTTTTTCTCGATATCGACTGGCAGGGCGCGCAGCAAATTCGCCAGAAGA
TGCCGCACGCGCGGAGTATCTTTATTTTACCGCCGTCTAAAATTGAACTGGACCGCCGTCTACGCGGTC
GCGGTCAGGACAGCGAAGAGGTCATTGCAAAGCGTATGGCGCAAGCTGTTGCAGAAATGAGCCATTA
CGCCGAATATGATTACCTGATTGTGAATGATGACTTCGATACCGCGTTGACCGATTTGAAGACCATTAT
TCGCGCCGAACGTCTGCGCATGAGCCGCCAAAAGCAGCGTCATGACGCTTTAATCAGCAAATTGTTGG
CAGAC*CACCACCACCACCACCAC*TGA

tmk-*7xHis*

ATGCGCAGTAAGTATATCGTCATTGAGGGGCTGGAAGGCGCAGGCAAAACTACCGCGCGTAATGTGG
TGGTTGAGACGCTCGAGCAACTGGGTATCCGCGACATGGTTTTCACTCGGGAACCTGGCGGTACGCAA
CTTGCCGAAAAGTTAAGAAGCCTGGTGCTGGATATCAAATCGGTAGGCGATGAAGTCATTACCGATAA
AGCCGAAGTTCTGATGTTTTATGCCGCGCGCGTTCAACTGGTAGAAACAGTCATCAAACCTGCGCTGG
CTAACGGCACCTGGGTGATTGGCGATCGCCACGATCTCTCCACTCAGGCGTATCAGGGCGGCGGACGT
GGTATTGACCAACATATGCTGGCAACACTGCGTGATGCTGTTCTCGGTGATTTTCGCCCCGACTTAACG
CTCTATCTCGATGTTACCCCGGAAGTTGGCTTAAAACGCGCGCGTGCGCGCGGCGAGCTGGATCGTAT
TGAGCAAGAGTCTTTCGATTTCTTTAATCGCACCCGCGCCCGCTATCTGGAACTGGCAGCACAAGATA
AAAGCATTCATACCATTGATGCCACCCAGCCGCTGGAGGCCGTGATGGATGCAATCCGCACTACCGTG
ACCCACTGGGTGAAGGAGTTGGACGCA*CACCACCACCACCACCACCAC*TGA

ndk-*5xHis*

ATGGCTATTGAACGTACTTTTTCCATCATCAAACCGAACGCGGTAGCAAAAAACGTCATTGGTAATAT
CTTTGCGCGCTTTGAAGCTGCAGGGTTCAAAATTGTTGGCACCAAAATGCTGCACCTGACCGTTGAAC
AGGCACGTGGCTTTTATGCTGAACACGATGGAAAACCGTTCTTTGATGGTCTGGTTGAATTCATGACCT
CTGGCCCGATCGTGGTTTCCGTGCTGGAAGGTGAAAACGCCGTTCAGCGTCACCGCGATCTGCTGGGC
GCGACCAATCCGGCAAACGCACTGGCTGGTACTCTGCGCGCTGATTACGCTGACAGCCTGACCGAAAA
CGGTACCCACGGTTCTGATTCCGTCGAATCTGCCGCTCGCGAAATCGCTTATTTCTTTGGCGAAGGCGA
AGTGTGCCCGCGCACCCGT*CACCACCACCACCAC*TGA

Enzyme Kinetics Dependence on Magnesium Concentration

Magnesium is a co-factor which is necessary for the activity of nucleotide kinase enzymes. The dependence of adenylate kinase reaction velocity on magnesium concentration was tested as described in the body of the text with the pyruvate kinase-lactate dehydrogenase coupled spectrophotometric assay. The final reaction buffer consisted of 50 mM potassium acetate, 20 mM Tris-acetate, 100 ug/mL BSA, 3 mM PEP, 2 mM ATP, 0.3 mM NADH, 1.25 U/mL pyruvate kinase, 2.25 U/mL lactate dehydrogenase, 0.0025 mg/mL adenylate kinase, 300 µM dAMP, and varied concentrations of magnesium acetate ranging from 1 – 20 mM. Solutions were brought up to 37°C before adding the substrate. Absorbance at 340 nm was read immediately after addition of substrate and followed for 5-10 minutes[1].

The $V_{max,\,app}$ in these reaction conditions at 300 µM dAMP was found to be 18.96 ± 3.52 µmole/min/mg protein, which is consistent with the V_o for 300 uM dAMP calculated using the experimentally determined V_{max} and K_M of adenylate kinase.

$$V_O = \frac{V_{max} * [S]}{K_M + [S]} = \frac{47.85 * 300}{359 + 300} = 21.78\ \mu mol/min/mg\ protein$$

The $K_{M,\,app}$ for Mg^{2+} was found to be 5.26 ± 1.21 mM, which is strikingly similar to the dissociation constant of Mg^{2+}•AK (K_d = 4.0 ± 1.5 mM) reported by Tan et al.[2] The working concentration of magnesium acetate in gDNA digestion reactions and in-house dNTP synthesis reactions was 10 µM, which is above the K_M^{Mg2+}, and thus does not limit the reaction velocity. However, other ions, such as potassium, are also reported to have an effect on enzyme activation and activity. Oeschger found that K^+ enhances the V_{max} of guanylate kinase while greatly reduced the K_M, and therefore increased affinity of the protein for dGMP[3]. However, the buffer used in the dNTP synthesis reactions is optimized for 100% activity of the DNA digesting enzymes. Although the activity of these nucleotide kinases is reduced in this buffer relative to previously published values, it is still sufficient to complete the conversion of dNMPs to dNTPs.

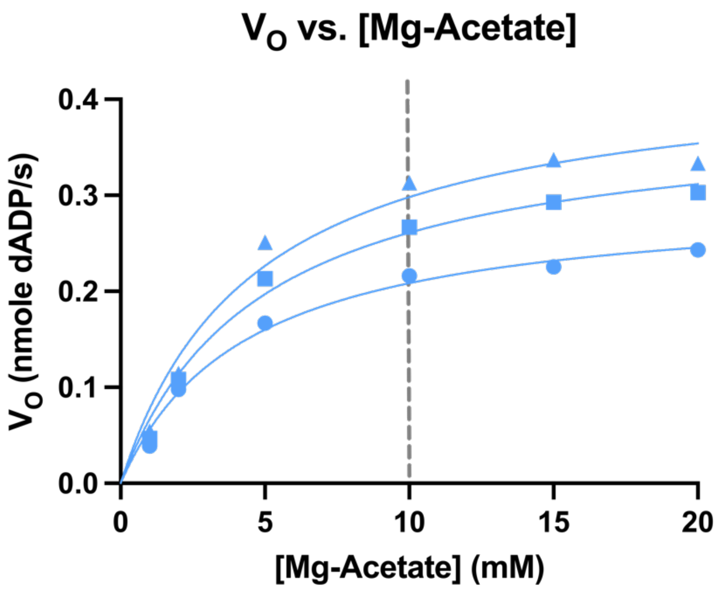

Figure S2. Adenylate kinase reaction velocity dependence on magnesium acetate concentration. Dashed line indicates the working concentration of magnesium used in all other enzyme kinetics experiments, gDNA digest, and dNTP synthesis reactions.

Buffer Composition of Enzyme Kinetics Reactions

Table S3. Buffer composition of enzyme kinetics reactions in this study compared to previously published studies of nucleotide monophosphate kinases.

	Tris	Magnesium	Potassium	ATP
CutSmart Buffer (this study)	20 mM Tris-acetate	10 mM MgAcetate	50 mM KCl	2 mM
Girons 1987 (adk)	50 mM Tris-HCl	2 mM $MgCl_2$	100 mM KCl	1 mM
Bucurenci 1996 (cmk)	50 mM Tris-HCl	2 mM $MgCl_2$	50 mM KCl	1 mM
Oeschger 1978 (gmk)	100 mM Tris-HCl	20 mM $MgCl_2$	240 mM KCl	5 mM
Munier-Lehmann 2001 (tmk)	50 mM Tris-HCl	2 mM $MgCl_2$	50 mM KCl	0.2-0.7 mM

Protein Extinction Coefficients

Table S4. Molecular properties of proteins calculated from the amino acid sequence using the Expasy Swiss Institute of Bioinformations ProtParam tool.

	ε_{molar}	MW	ε1%
adk-8xHis	10430	24683.14	4.225556
cmk-6xHis	8480	25569.19	3.316491
gmk-6xHis	17420	24415.56	7.134794
tmk-7xHis	18450	24743.13	7.744640
ndk-5xHis	4470	16149.15	2.767948

According to the NanoDrop user manual, the protein concentration can be calculated as

$$C = \frac{A_{280}}{\varepsilon 1\%} * 10$$

Where C is the concentration in mg/mL, A_{280} is the absorbance at 280 nm, 10 is a correction factor to convert from g/100 mL to mg/mL, and ε1% is the percent extinction coefficient and is equal to

$$\varepsilon 1\% = \frac{\varepsilon_{molar}}{\text{MW}} * 10$$

where ε_{molar} is the molar extinction coefficient.

Enzyme kinetics experiments were initially conducted using the assumption 1 absorbance at A280 = 1 mg/mL and was later corrected as detailed in Table S5.

Table S5. Protein concentrations in enzyme kinetics reactions calculated from A_{280} nm and extinction coefficients.

Protein	A280 nm Reading*	Concentration (mg/mL)	Protein added to reaction solution (µL)	Final volume of reaction solution (µL)	Final protein concentration (mg/mL)
Adk	1.00	2.37	12.5	5000	0.00592
Cmk	1.05	3.17	12.5	5000	0.00792
Gmk	1.01	1.40	12.5	5000	0.00350
Tmk	1.00	1.34	12.5	5000	0.00335
Ndk	0.80	2.89	16.0	5000	0.00925

*proteins were intentionally diluted to A_{280} nm ~1.00 for storage

The specific activity (V_{max}) of the protein in µmol/min/mg protein is then calculated as

$$V_{max} = \frac{V \frac{\mu mol}{s}}{(C_{prot}\ mg/mL) * 0.2\ mL} * \frac{60\ s}{1\ \text{min}}$$

Where V is the maximum velocity, C is the concentration of the protein in the reaction solution in mg/mL, 0.2 is the volume of the reaction in mL, and 60 is the conversion factor between seconds and minutes.

Lineweaver Burk Plots – NMP Kinases

Enzyme kinetics parameters were calculated using both nonlinear regression curve fit with the Michaelis Menten equation and linear regression curve fit with Lineweaver Burk equation. The Lineweaver Burk plot often compound or distort errors in the data, and thus are deemed less accurate for calculating kinetic parameters than a direct curve fit with Michaelis Menten. For this reason, we have presented the parameters calculated from Michaelis Menten in the body of the text. However, we have also included a summary of the Lineweaver Burk analysis below, whose results are consistent with the nonlinear regression parameters.

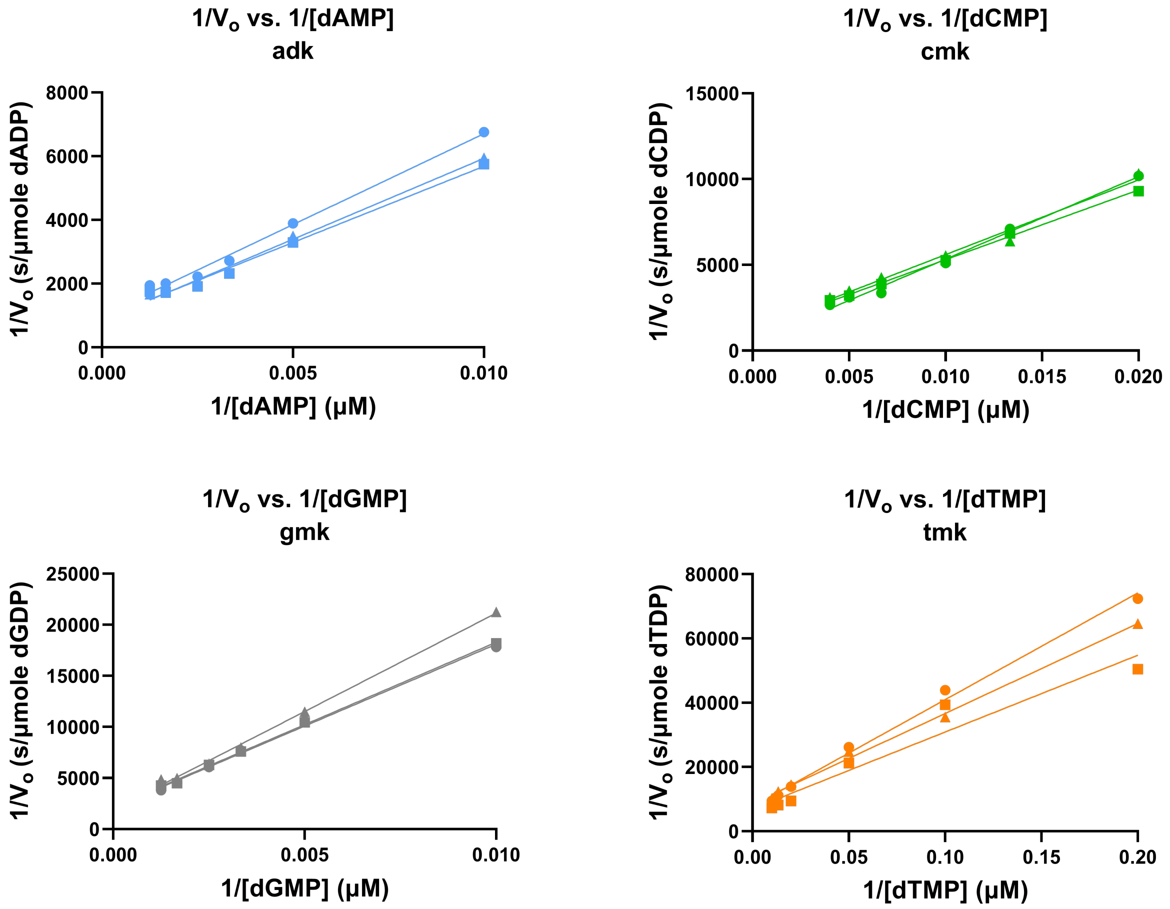

Figure S3. Lineweaver-Burk plots of inter-batch variation of NMP kinase activity.

Kinetic parameters, maximum velocity V in μmol/s and affinity constant K_M, are calculated from the slope and y-intercept outputs from linear regression of Lineweaver-Burk plots as described in the equations below. The specific activity (V_{max}) is calculated as described previously. The protein concentrations for these experiments were quantified using NanoDrop A_{280} nm readings.

$$\frac{1}{V_O} = \frac{K_M}{V * [S]} + \frac{1}{V}$$

$$V = \frac{1}{\text{y-intercept}}$$

$$K_M = V * \text{slope}$$

NMP Kinases – Batch to Batch Variation

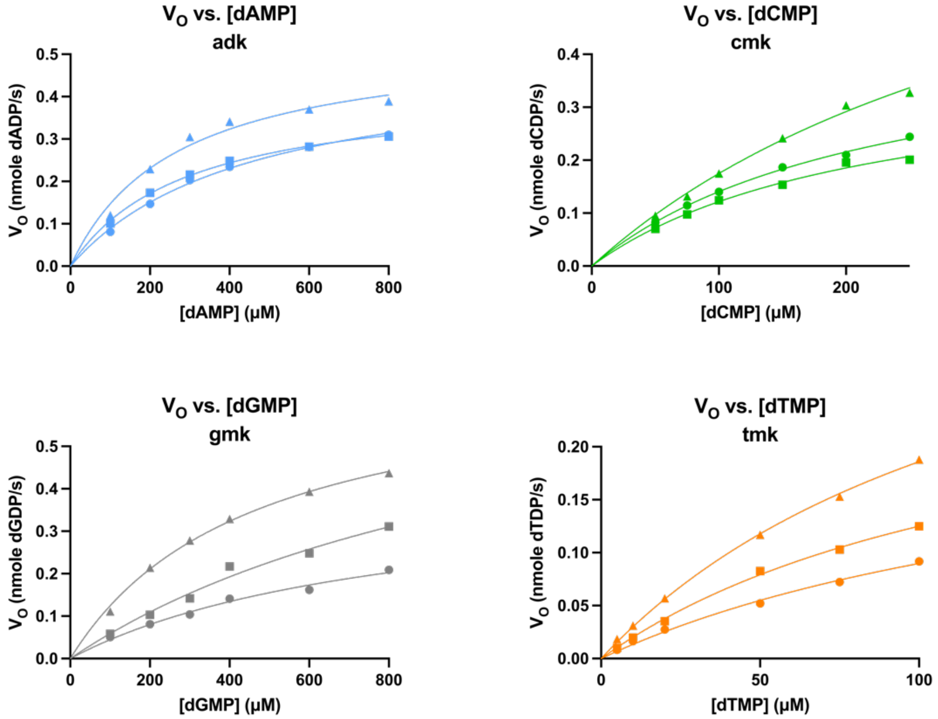

Figure S4. Michaelis-Menten curves of independent batches of NMP kinase proteins (n=3). Protein concentration quantified with Bradford assay.

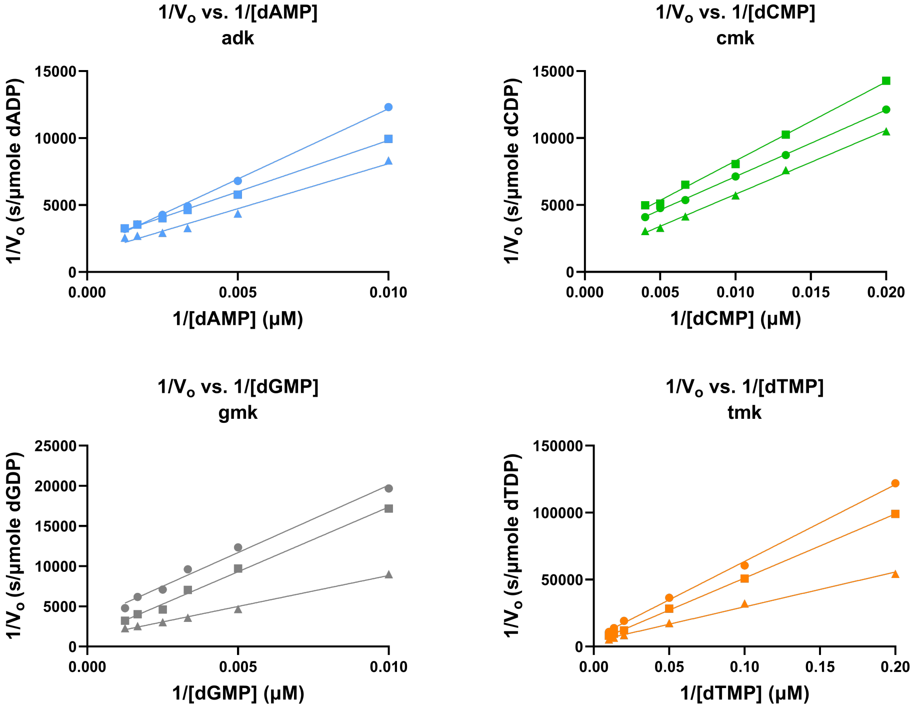

Figure S5. Lineweaver Burk plots of independent batches of NMP kinase proteins (n=3). Protein concentration quantified with Bradford assay.

Kinetic Parameter Comparison

As expected, inter-batch variation is smaller than batch to batch variation. Regardless of protein quantitation with NanoDrop or Bradford assay and protein batch, kinetic parameters are on the same order of magnitude. Kinetic parameters calculated by Lineweaver Burk or by Michaelis-Menten give similar values. Values reported in the body of the text are from nonlinear regression with the Michaelis-Menten curve, as it is deemed more accurate due to the high weighting Lineweaver-Burk places on substrate concentrations below the K_M.

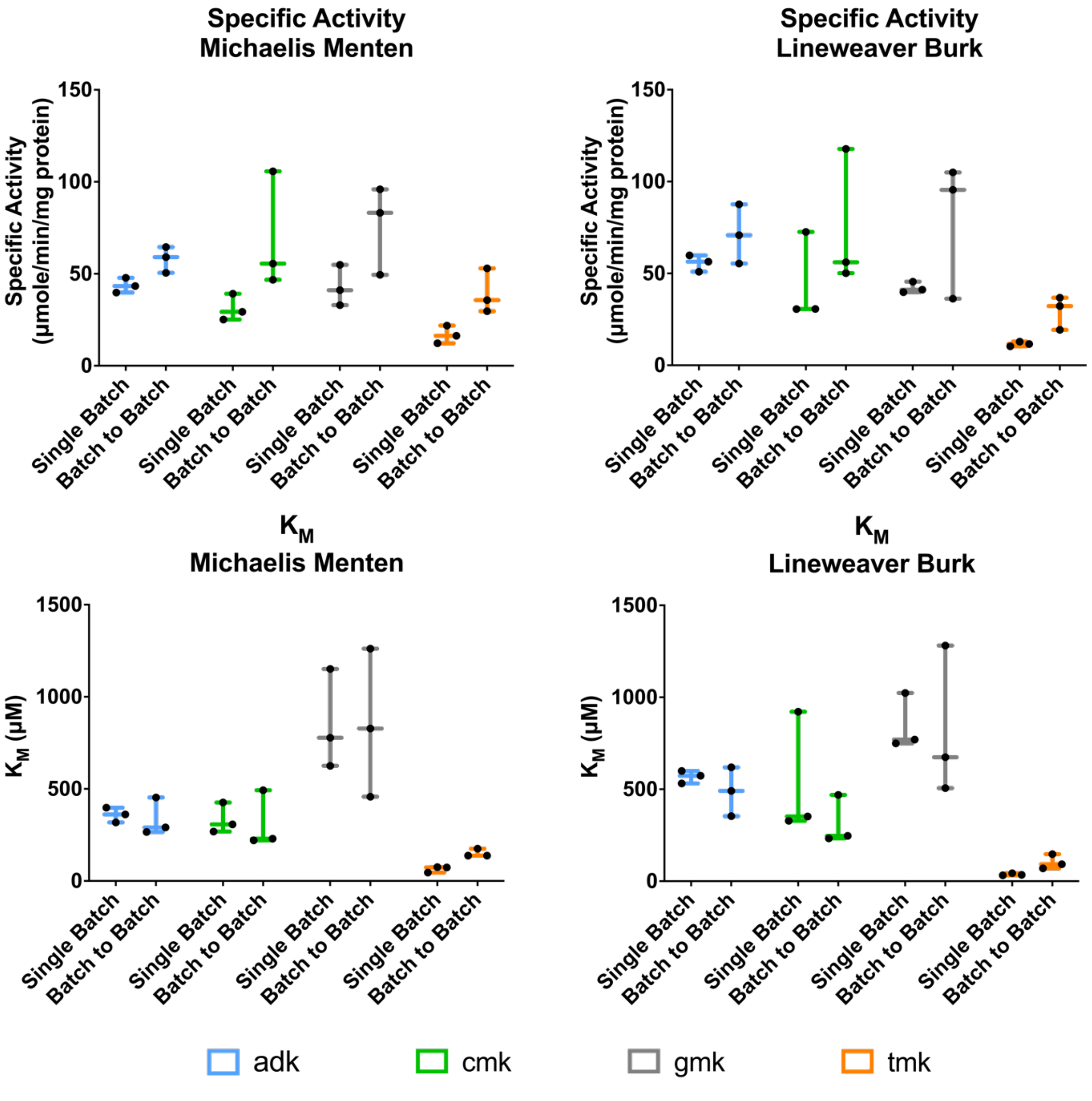

Figure S6. Comparison of specific activity and K_M values calculated for the proteins using different regression methods. Single batch refers to one batch of protein performed in triplicate. Single batch protein concentration was quantified using A_{280} nm and protein coextinction coefficients. Batch to batch refers to three independent batches of protein each performed in a single replicate. Batch to batch protein concentrations were quantified using Bradford assay.

Lineweaver Burk Plots – Nucleotide Disphosphate Kinase

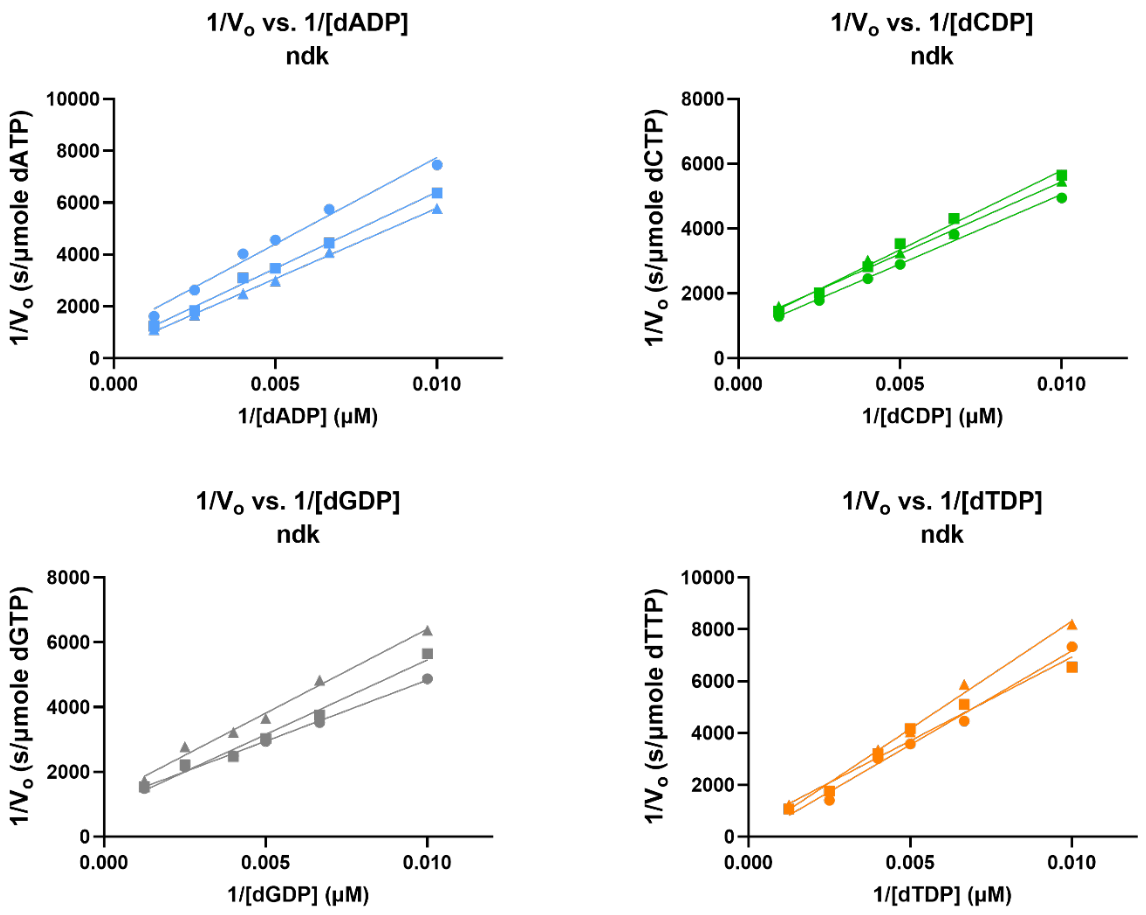

Figure S7. Lineweaver Burk plots of inter-batch variation of NDK activity toward the four target dNDP substrates. Protein concentration quantified with NanoDrop.

Nucleotide Diphosphate Kinase – Batch to Batch Comparison

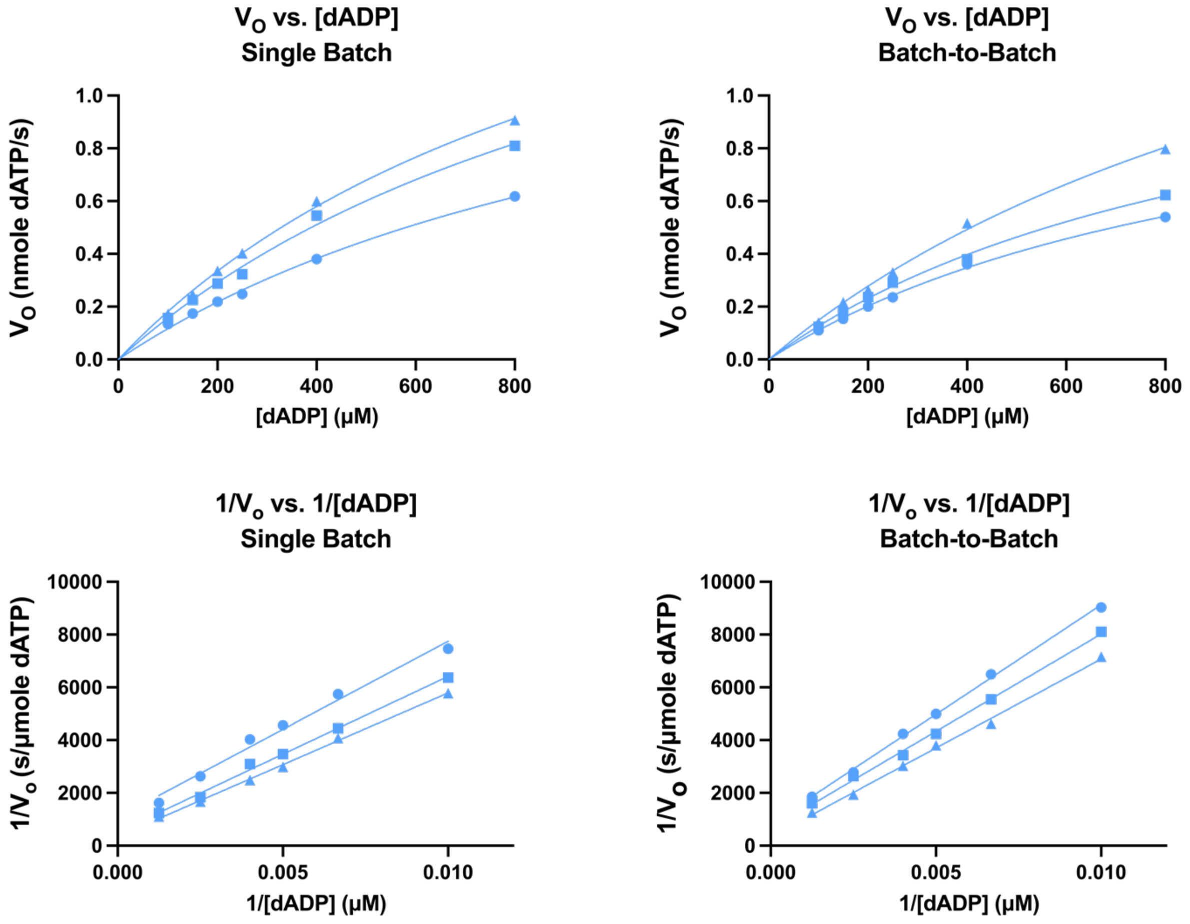

Figure S8. Inter-batch and batch to batch variation of NDK activity toward dADP substrate. For the batch-to-batch kinetics calculated by nonlinear regression with Michaelis-Menten, $V_{max,dADP}$ = 193.12 ± 60. 88 µmol/min/mg protein and $K_{M,dADP}$ = 1138 ± 206 µM. For batch-to-batch kinetics calculated by linear regression with Lineweaver-Burk, $V_{max,dADP}$ = 232.18 ± 116.71 µmol/min/mg protein and $K_{M,dADP}$ = 1400 ± 567 µM.

The difference between the single batch specific activity reported in the body of the text and the batch-to-batch specific activity presented here is largely due to the difference in protein quantitation method. The single batch protein concentration was quantified using A280 nm and coextinction coefficients, and the batch-to-batch protein concentration was quantified using Bradford assay. While the A_{280} nm and Bradford concentrations were consistent for the NMP kinases, there appears to be a discrepancy between the quantitation methods for NDK.

Kinetics of Unpurified Kinase Lysate

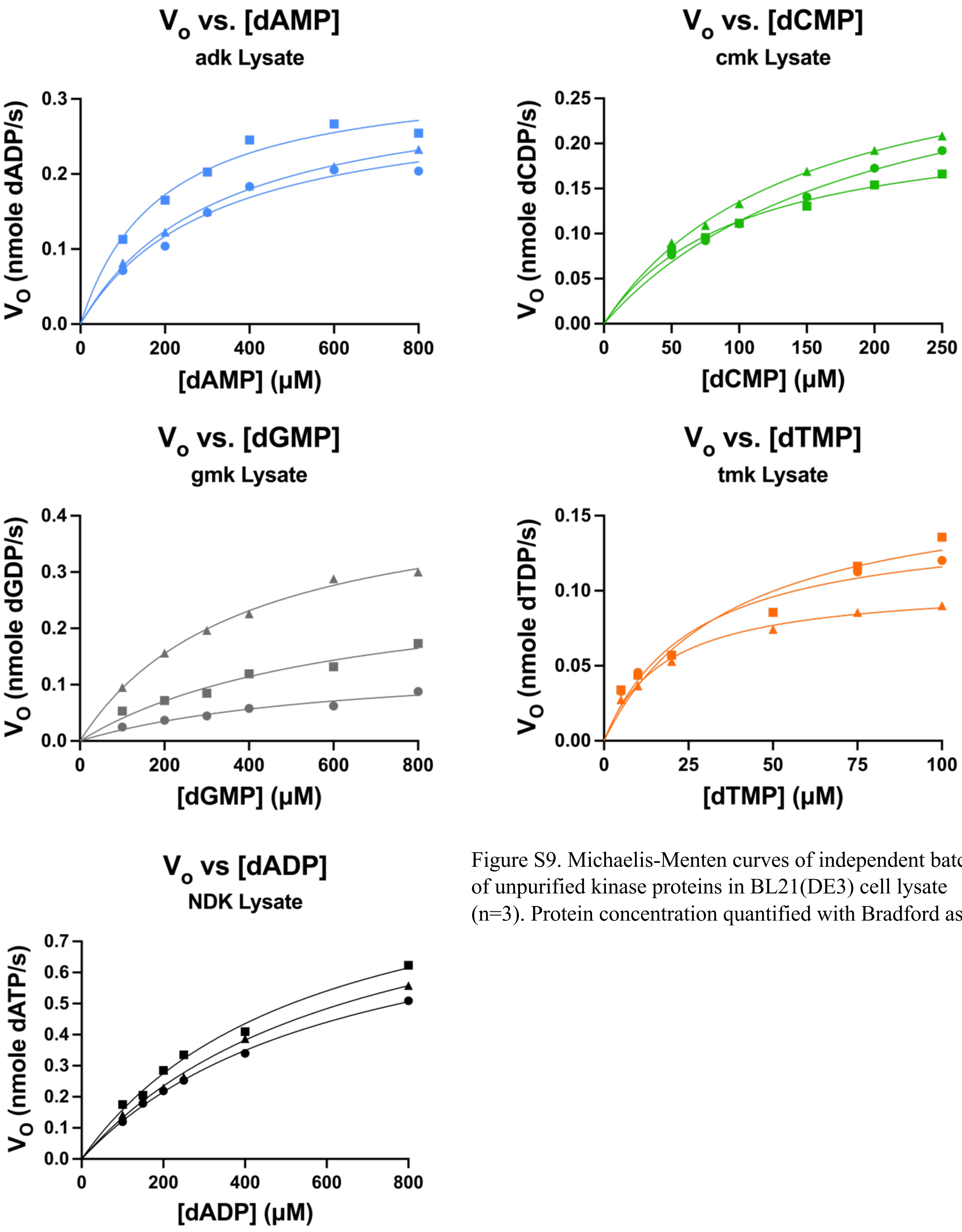

Figure S9. Michaelis-Menten curves of independent batches of unpurified kinase proteins in BL21(DE3) cell lysate (n=3). Protein concentration quantified with Bradford assay.

Table S6. Specific activity values for purified protein and unpurified protein in cell lysate calculated from Michaelis-Menten nonlinear regression.

Purified Protein Specific Activity (umole/min/mg protein)	Lysate Specific Activity (umole/min/mg protein)	Fold difference in activity
Vmax * 60 /(Protein conc * volume)	Vmax * 60 /(Protein conc * volume)	(Purified Protein Specific Activity) (Lysate Specific Activity)
59.02	36.17	1.63
50.47	40.31	1.25
64.54	39.12	1.65
		Average: 1.51
55.524	41.00	1.35
46.752	27.55	1.70
105.696	39.40	2.68
		Average: 1.91
49.452	17.42	2.84
95.88	35.34	2.71
83.148	54.36	1.53
		Average: 2.36
29.652	17.57	1.69
35.652	21.00	1.70
52.92	12.55	4.22
		Average: 2.53
148.32	110.33	1.34
168.6	125.52	1.34
262.44	123.72	2.12
		Average: 1.60

The pure protein specific activity is paired with the corresponding batch of unpurified lysate from which it was purified. The pure protein specific activity is calculated from the nonlinear regression curve fits in Figure 4, and the lysate specific activity is calculated from the nonlinear regression curve fits in Figure S9.

Negative Control BL21(DE3) Lysate Kinetics

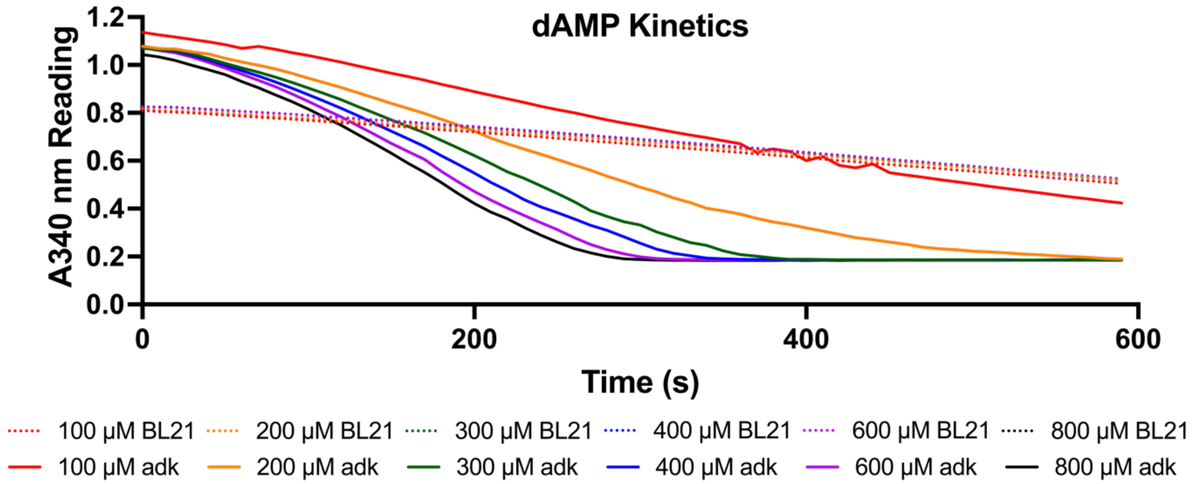

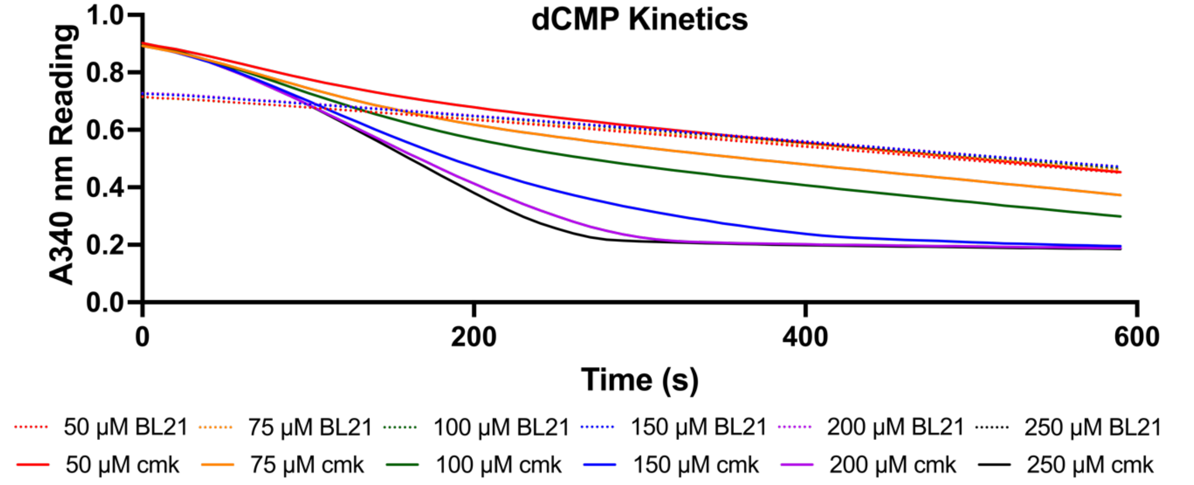

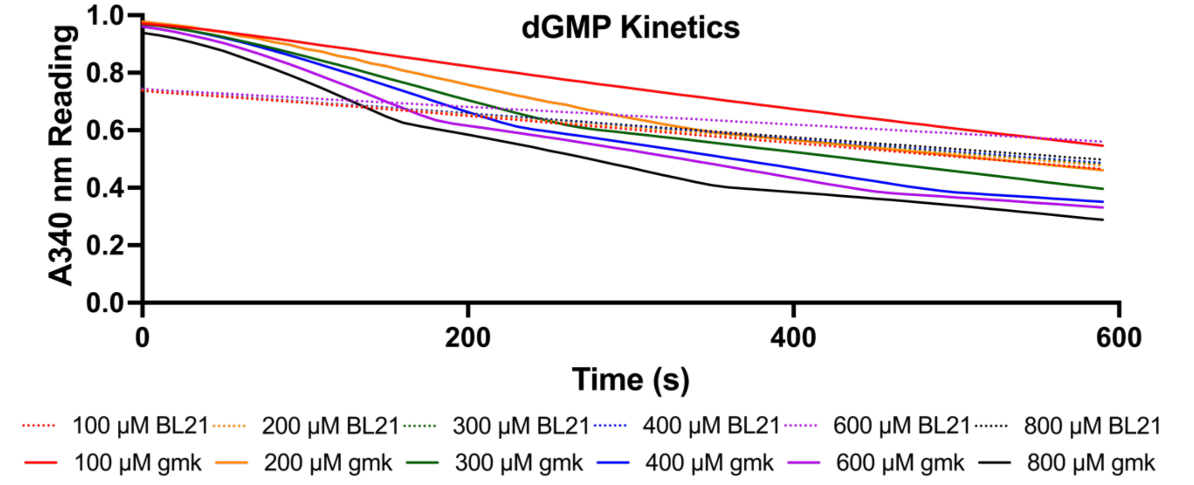

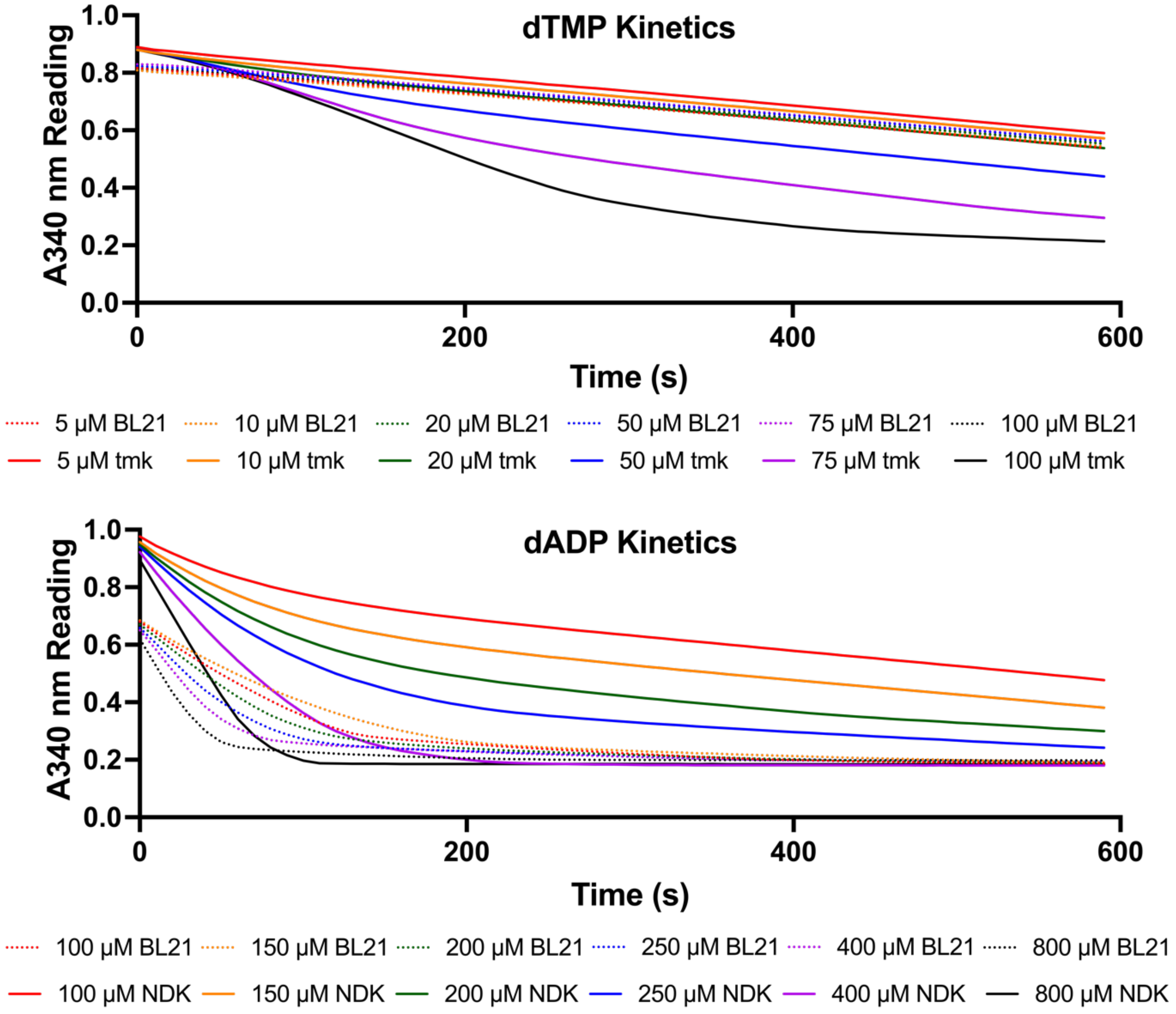

Figure S10. Raw A340 nm readings from kinetics assays of BL21(DE3) negative control lysate, which does not contain any overexpressed kinases (BL21 in dashed lines) compared to the readings from kinetic assays of BL21(DE3) lysate containing the overexpressed kinase listed (solid line). adk: adenylate kinase. cmk: cytidylate kinase. gmk: guanylate kinase. tmk: thymidylate kinase. NDK: nucleotide diphosphate kinase.

The kinetics assay follows the absorbance of NADH at 340 nm over time. The double coupled pyruvate kinase – lactate dehydrogenase consumes NADH at a 1:1 molar ratio with the amount of product produced by the nucleotide kinase. A calibration curve of molar NADH standards is used to calculate the moles of NADH at each time point, then the moles of NADH consumed, and finally the reaction velocity for each substrate concentration. Given that the BL21(DE3) negative control samples showed minimal variation in reaction velocity for different substrate concentrations (apart from dADP substrate), no kinetics analysis was performed on the negative control samples.

SDS-PAGE Image Analysis

To quantify protein purity from SDS-PAGE images, the image was imported into ImageJ and the image was black/white inverted. A rectangular region of interest (ROI) was drawn around the entirety of the induced protein lane (Figure 4). The lane intensity was quantified with the ImageJ Integrated Density function. An ROI of the same size was drawn around the purified protein lane, and the integrated density was again measured. This process was repeated with a smaller ROI to quantify the intensity of the kinase band in both the induced and purified lanes. The relative purity was calculated by dividing the integrated density ratio of kinase band : total lane for the purified lane by the integrated density ratio of kinase band : total lane for the induced lane.

Table S7. Results of SDS-PAGE image analysis.

**Protein**	**SDS-PAGE Quantification of Purity (n = 1)**
adk	2.23
cmk	1.61
gmk	1.89
tmk	1.71
ndk	2.42

HPLC dNMP Standard Curves

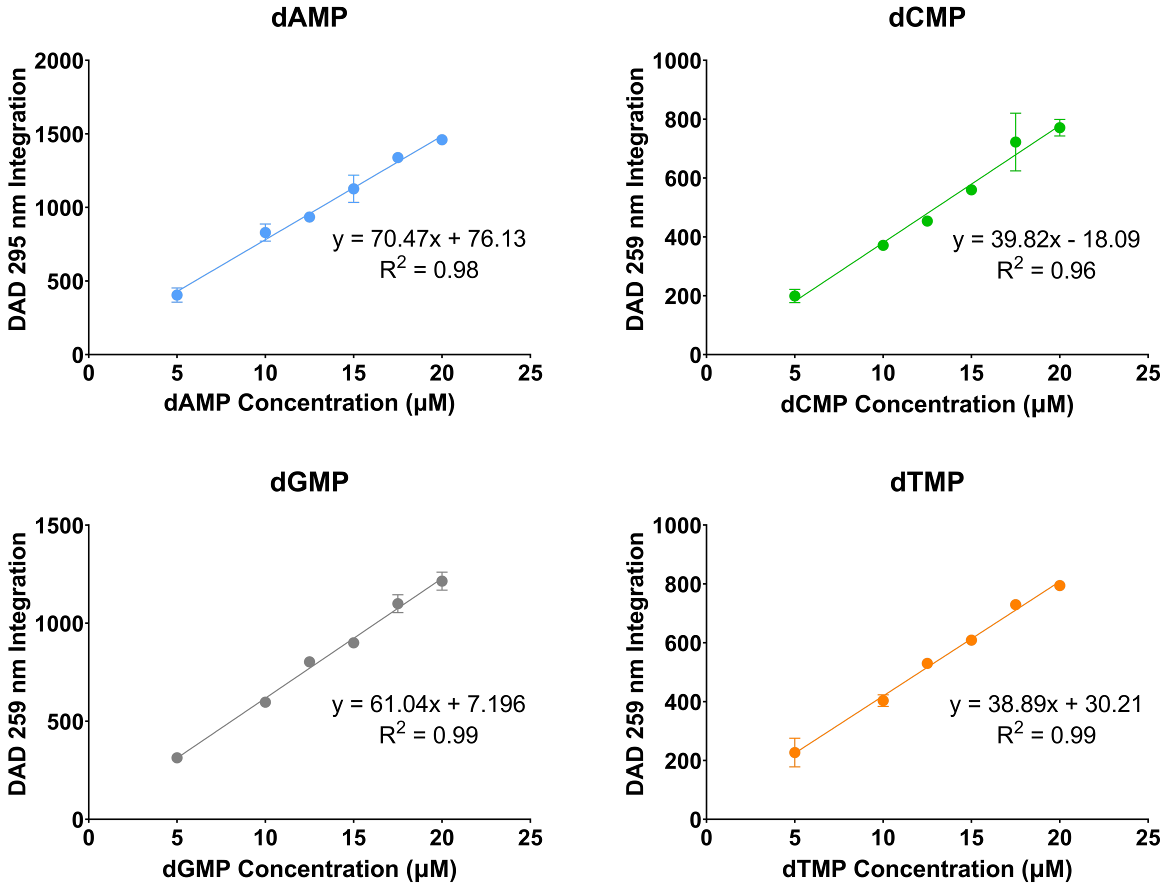

Figure S11. Concentration calibration curves for dNMP standards on HPLC (n = 3).

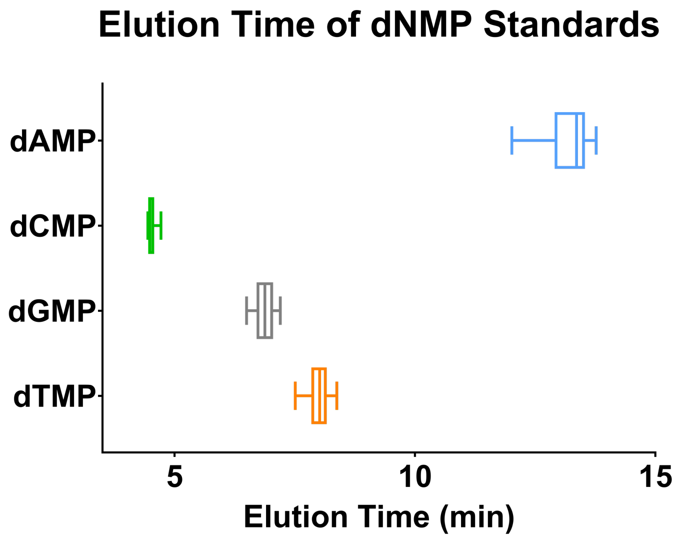

Figure S12. Variation in elution time of dNMP standards on HPLC (n = 18). Box plot showing mean and standard deviation with whiskers at minimum and maximum values.

HPLC dNTP Standard Curves

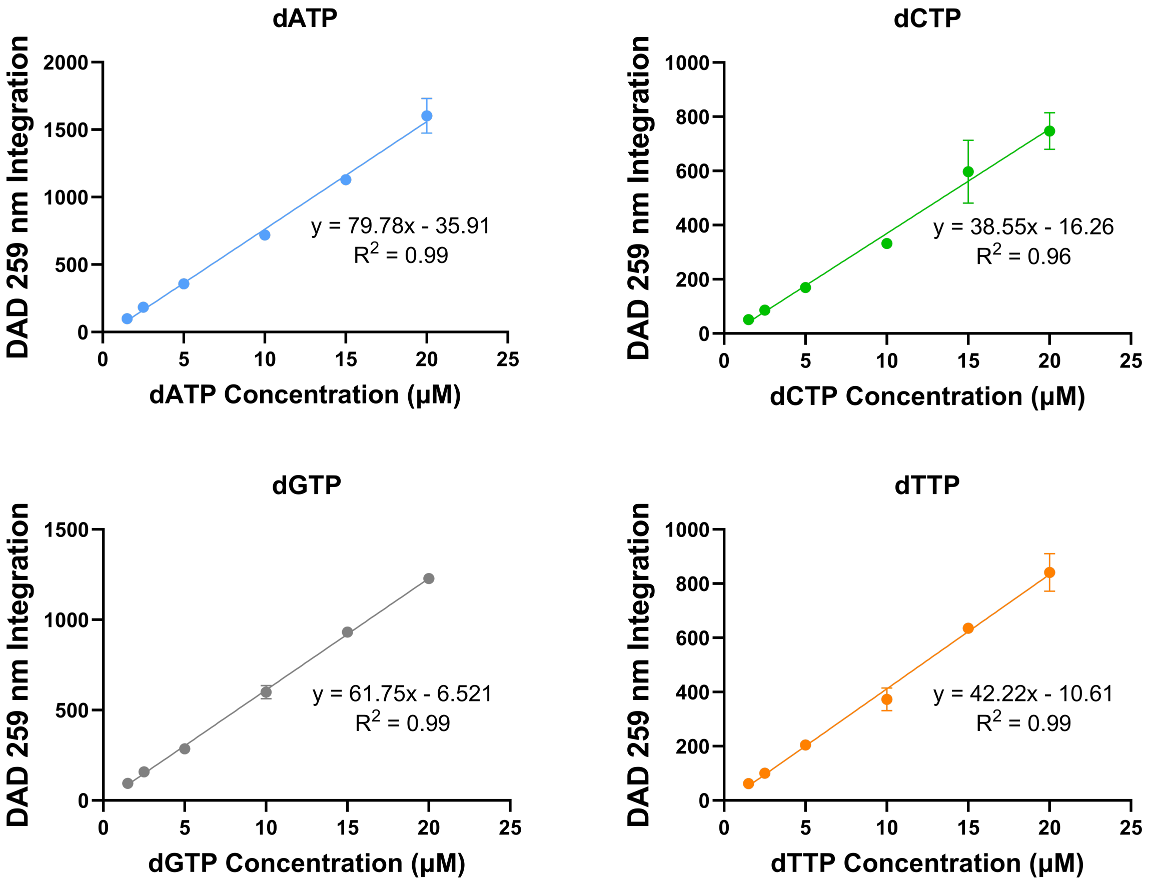

Figure S13. Concentration calibration curves for dNTP standards on HPLC (n = 3).

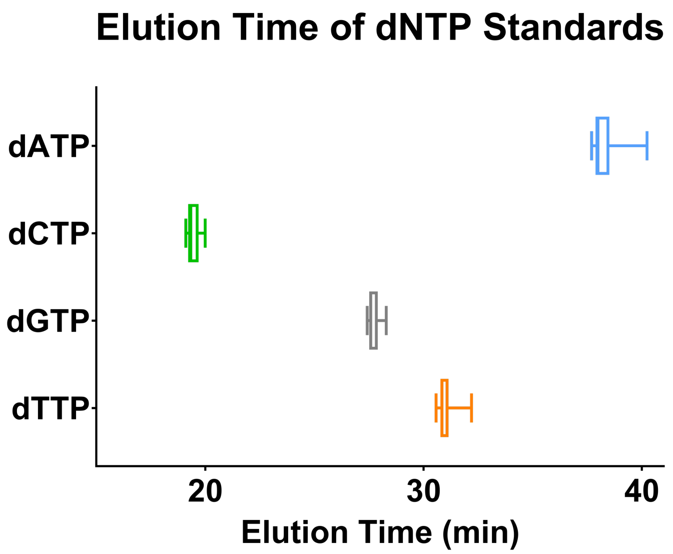

Figure S14. Variation in elution time of dNTP standards on HPLC (n = 18). Box plot showing mean and standard deviation with whiskers at minimum and maximum values.

HPLC ADP and ATP Elution Time

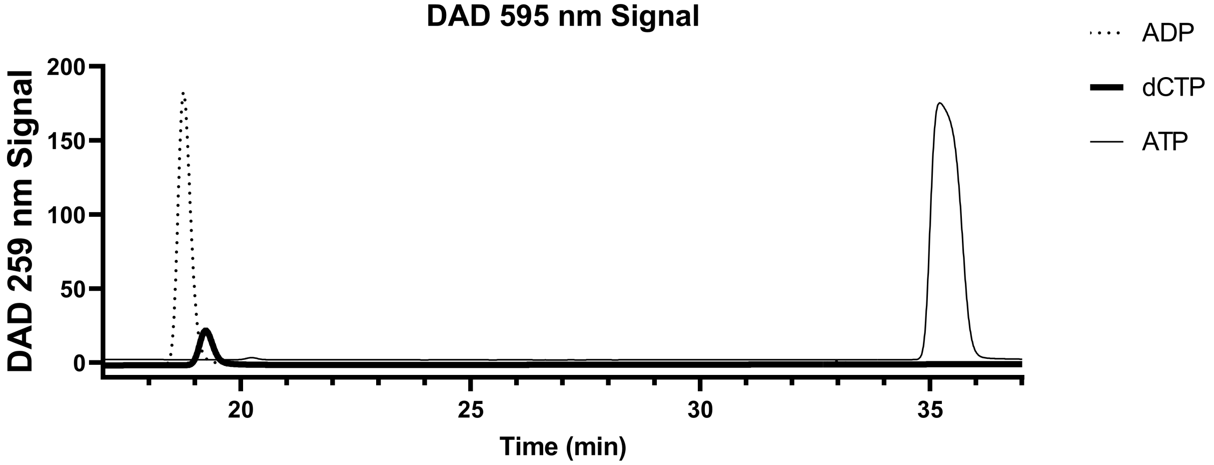

Figure S15. Co-elution of ADP and dCTP peaks. As noted by Fehlau et al., ATP and dCTP elute at a similar time on this HPLC method and are often unable to be resolved[4]. Other methods including LC/MS and MS of HPLC fractions were attempted but also failed to resolve the compounds. Other LC-MS methods have been published but report co-elution of ATP and dGTP[5]. Due to the similarity in molecular structure, charge, and weight of the nucleotides, they are difficult to separate chromatographically.

Unlike Fehlau et al., the dCTP concentration cannot be deduced from the remaining concentration of dCMP and dCDP because the dCDP peak is also not well resolved from other contaminants and nucleotides in our reaction mix. In some cases, the ADP and dCTP peaks could be resolved. In other cases, a distinct shoulder was observed on the ADP peak, and the dCTP concentration was calculated using OriginLab peak splitting algorithms as shown in Figure S14. The algorithm identifies hidden peaks by calculating the second derivative to determine local extrema in the data.

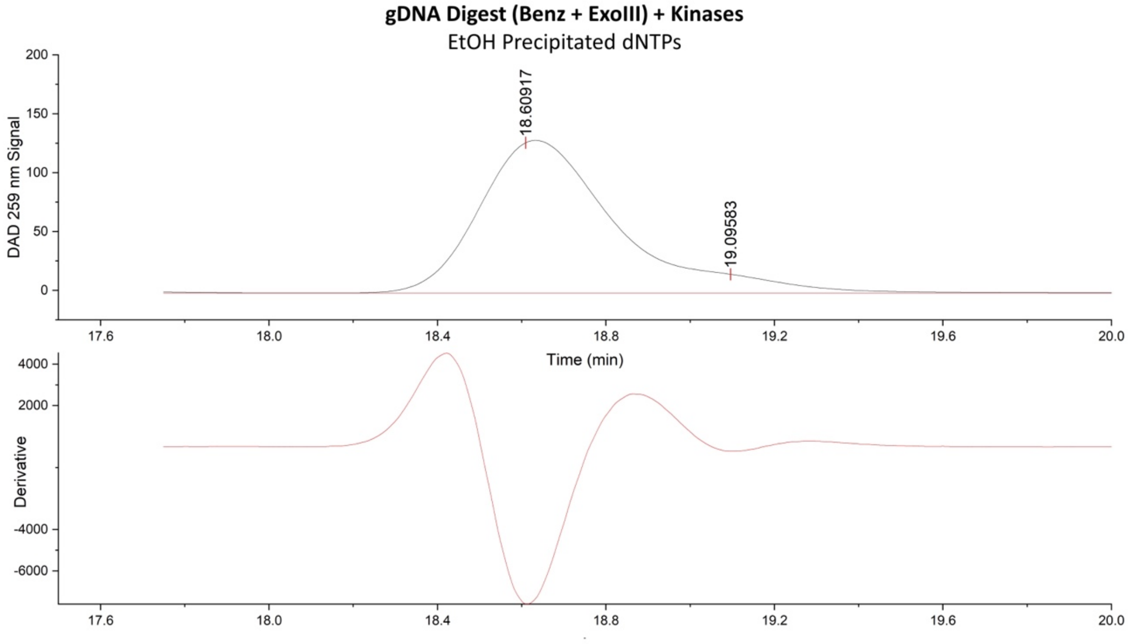

Figure S16. Second derivative hidden peak finder algorithm applied to HPLC spectrum of in-house dNTPs. In-house dNTPs synthesized from a 60 µg gDNA digest with Benzonase and Exonuclease III, followed by incubation with kinase enzymes and ATP at 37°C for 30 min. In-house dNTPs precipitated with EtOH and resuspended in 562.5 uL water (30x dilution factor) before loading on HPLC.

Lambda DNA PCR

Table S8. Primers used to amplify lambda DNA genome in PCR

Expected Size	Forward Primer	Reverse Primer	Annealing Temp	Extension Time
0.5 kb	GGGATATCATCAAAGCCATGA ACAAAGCAGCC	GAAACGCTTCATGGTGAG CGTGTTATCCC	62°C	30 s
1.0 kb	GGGATATCATCAAAGCCATGA ACAAAGCAGCC	CCAGATGTGAAAGGTCAC ACTGTCAGGTG	62°C	3 min
2.0 kb	GGGATATCATCAAAGCCATGA ACAAAGCAGCC	CTTCGTCCGTCTTTCTGTA CTGTTGCCAC	62°C	3 min
3.0 kb	GGGATATCATCAAAGCCATGA ACAAAGCAGCC	CGCTGCTCCTGACTGTTCG CG	62°C	3 min
5.0 kb	GGGATATCATCAAAGCCATGA ACAAAGCAGCC	GCATCAGACGATCCAGCG CAGTGTC	62°C	7 min 30 s
7.5 kb	GGGATATCATCAAAGCCATGA ACAAAGCAGCC	GCTTGATTACGGGCAAAT CCCCCC	62°C	7 min 30 s

Table S9. PCR reaction mixes

	Commerical dNTPs Positive Control	Commercial dNTPs Negative Control	In-house dNTPs Positive Control	In-house dNTPs Negative Control
10 uM Forward Primer	1.25 µL	1.25 µL	1.25 µL	1.25 µL
10 uM Reverse Primer	1.25 µL	1.25 µL	1.25 µL	1.25 µL
50 ug/mL Lambda DNA	1 µL	0 µL	1 µL	0 µL
10X ThermoPol Buffer	2.5 µL	2.5 µL	2.5 µL	2.5 µL
100 mM MgSO4	1.5 µL	1.5 µL	0 µL	0 µL
10 mM Commerical dNTPs	0.5 µL	0 µL	0 µL	0 µL
Water	16.75 µL	17.75 µL	0 µL	1 µL
In-house dNTP reaction mix	0 µL	0 µL	18.75 µL	18.75 µL
DeepVent Polymerase	0.25 µL	0.25 µL	0.25 µL	0.25 µL
Total reaction volume	25 µL	25 µL	25 µL	25 µL

Kinase Knockout

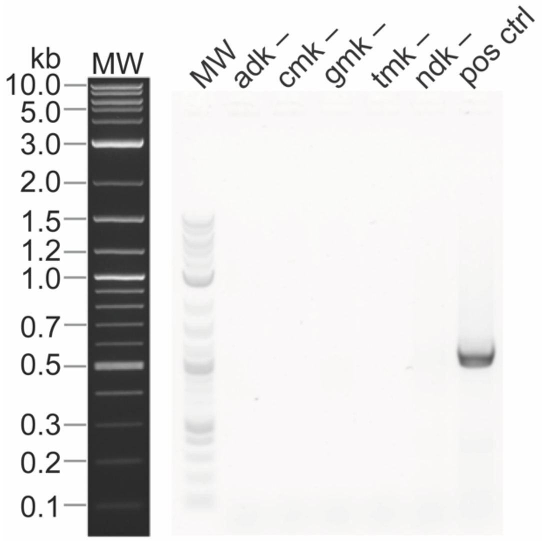

Figure S17. Results of 1 kb lambda DNA PCR using 18.75 µL in-house dNTPs synthesized from commercial dNMP standards. **MW:** 1 kb plus (NEB). **adk –** : no adenylate kinase added to dNTP reaction. **cmk –** : no cytidylate kinase added to dNTP reaction. **gmk –** : no guanylate kinase added to dNTP reaction. **tmk –** : no thymidylate kinase added to dNTP reaction. **ndk –** : no nucleotide diphosphate kinase added to dNTP reaction. **pos ctrl:** all NMP kinases and NDK included in dNTP reaction, 1% agarose. 90V, 30 min.

Effect of EtOH Precipitation on Synthesized dNTPs

Precipitation of dNTPs with ethanol (EtOH) allows the dNTPs to resuspended in a smaller volume of water, and thus are more concentrated, as indicated by the increased band brightness in lane 3, relative to lanes 1 and 2 in Figure S18.

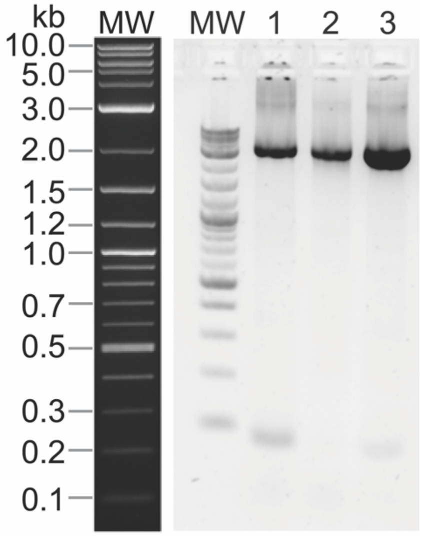

Figure S18. Results of 3 kb lambda DNA PCR with different preparations of in-house dNTPs. **MW:** 1 kb plus (NEB). **1:** 18.75 µL in-house dNTPs from commercial dNMPs (fresh). **2:** 18.75 µL in-house dNTPs from kinase reaction with commercial dNMPs, followed by EtOH precipitation and resuspension in 50 µL water. **3:** 18.75 µL in-house dNTPs from kinase reaction with commercial dNMPs, followed by EtOH precipitation and resuspension in 18.75 µL water. 2% agarose. 100V, 30 min.

PCR Yield

A PCR reaction was performed with 3 kb lambda genome primers using either 200 μM commercial dNTPs, 150 μM commercial dNTPs, or in-house dNTPs (estimated to have a concentration of approximately 150 μM), following the protocols described in the main body of the text. After thermocycling, the PCR reaction was diluted 1:400 and the yield was quantified with PicoGreen. The concentration of DNA was determined from a calibration curve with Lambda DNA. In-house dNTP samples were quantified with PicoGreen prior to the PCR reaction and blank subtracted from the final PCR concentration to account for the contribution of undigested DNA fragments toward background fluorescence. There were no statistically significant differences observed between any of the groups when using a Kruskal-Wallis test with multiple comparisons.

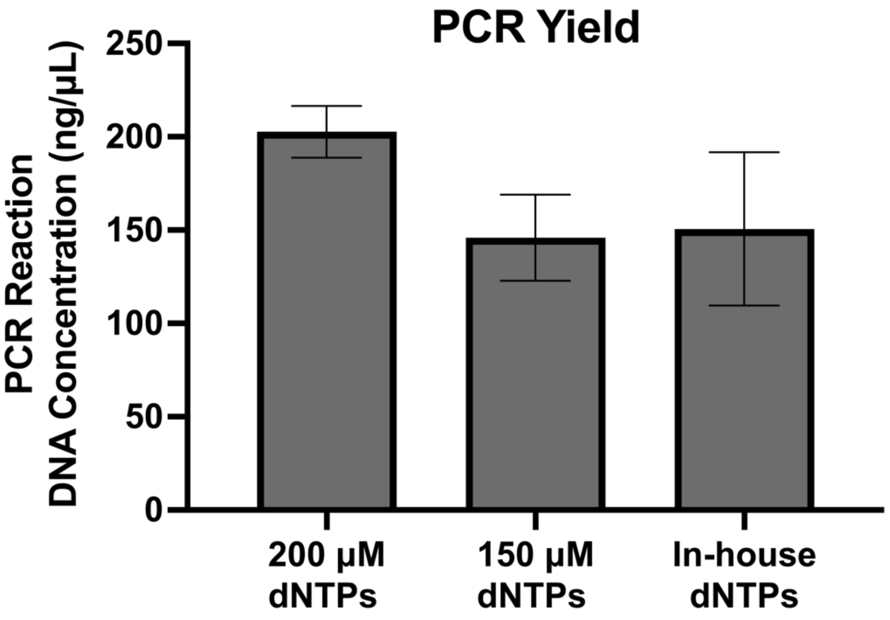

Figure S19. DNA concentration measured at the endpoint of PCR reactions with varied concentration of dNTPs. Error bars represent SEM. 200 μM and 150 μM dNTP samples contain commercial dNTPs. In-house dNTPs were produced from 60 μg gDNA and concentrated with ethanol precipitation.

E. coli genome PCR

A PCR was performed using primers to detect fragments of the E. coli genome to determine if contaminating DNA fragments in the in-house dNTPs produce false positive results. A PCR reaction mix was prepared as described in Table S9, using the adk forward and reverse primers listed in Table S2. 1 μL of 10 ng/μL adk-His-pET24a plasmid DNA was used in place of Lambda DNA as the template. For negative controls, 1 μL of water was added in place of the template. 30 cycles of PCR were performed with a 50°C annealing temperature and 40 s extension time.

To remove undigested DNA fragments, the dNTP reaction solution was added to a PCR clean-up column (NEB) with 350 μL of binding buffer immediately following incubation with kinases. The column was spun for 1 minute at 13000 rpm. The silica column was discarded, and the filtrate (total volume ~400 μL) was collected. The PCR clean-up kit used with a 7:1 binding buffer to sample ratio is optimized to remove any single stranded DNA larger than 200 basepairs. The silica column was thus expected to bind the undigested DNA fragments, while the dNTPs should pass into the flow through. The dNTPs were then recovered by ethanol precipitation. 37 μL of 3 M NaCl and 3 vols (1200 μL) of ice cold 100% ethanol were added to the PCR clean-up column flow-through. The ethanol solution was incubated 1 hour at -80C, then centrifuged 20 min at 4C. The supernatant was discarded, and the formed pellet was air dried for 30 min. The pellet was then resuspended in 18.75 μL of water and used immediately in the PCR reaction.

PCR using in-house dNTPs to target the *E. coli* genome resulted in false positive results. Attempts to purify the dNTPs using a PCR clean-up column resulted in failed PCRs for the positive control samples. The dNTPs were thus unable to be recovered from the column flow through.

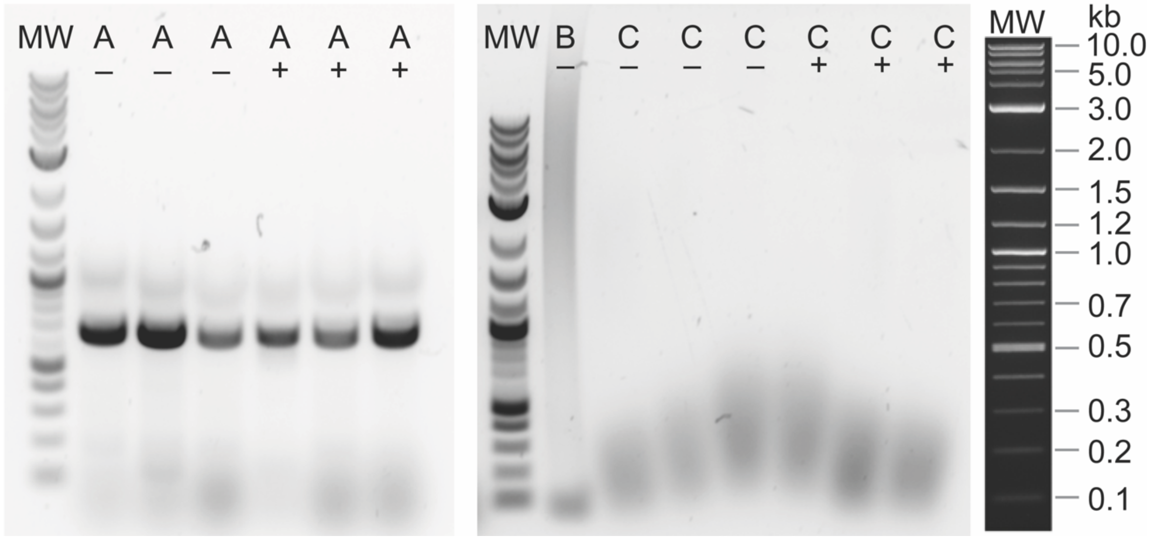

Figure S20. PCR with in-house dNTPs targeting the *E. coli* genome. "+" samples contain 10 ng adk-His-pET24a plasmid as template. "–" samples do not have any added template. A: in-house dNTPs synthesized from 60 ug gDNA. B: 200 uM commercial dNTPs. C: In-house dNTPs synthesized from 60 ug gDNA and purified with a PCR clean-up column. MW: 1 kb plus (NEB).

Increased Digestion Enzyme Concentration

Increasing the amount of digestion enzyme does not improve the yield of dNTPs or the ability to perform larger fragment PCR reactions.

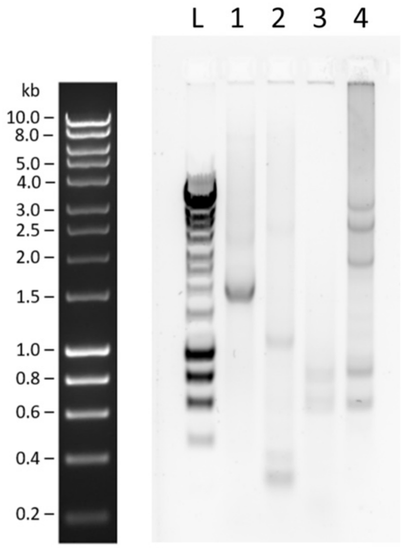

Figure S21. In-house dNTPs synthesized from a 60 µg gDNA digest with 16.75 U Benz and 500 U ExoIII, then applied in lambda gene PCR reactions (no ethanol precipitation). Lane 1: 2 kb lambda PCR with in-house dNTPs (16.75 U Benz, 500 U ExoIII). Lane 2: 5 kb lambda PCR with in-house dNTPs (16.75 U Benz, 500 U ExoIII). Lane 3: 7.5 kb lambda PCR with in-house dNTPs (16.75 U Benz, 500 U ExoIII). Lane 4: 7.5 kb lambda PCR reaction with 200 µM commercial dNTPs. L: 1 kb HyperLadder (Meridian Bioscience BIO33026).

dNTP Longevity

dNTPs are stable for one week when stored in precipitated form at 4C. dNTPs show moderate stability after one week stored in precipitated from at room temperature (RT).

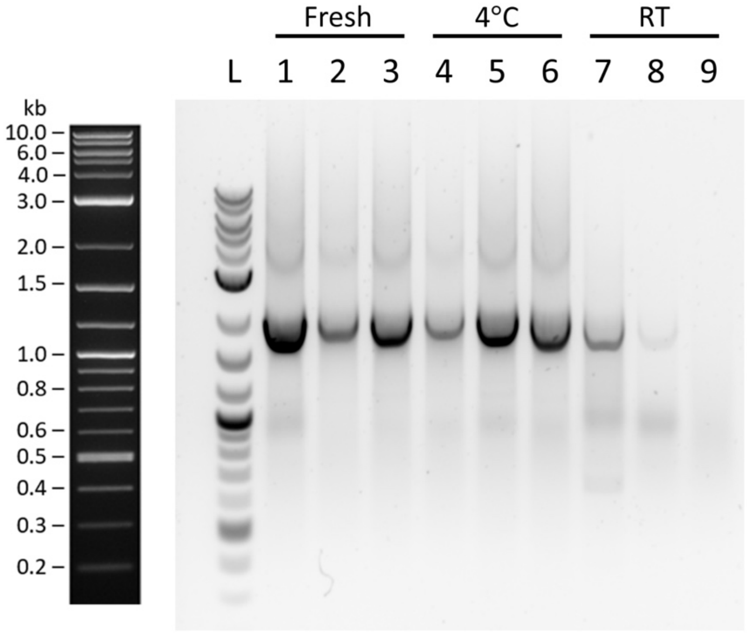

Figure S22. PCR of 2 kb lambda DNA fragment with in-house dNTPs stored under different conditions. Following dNTP synthesis, the dNTPs were precipitated with ethanol, then air dried. Fresh samples were resuspended in 18.75 µL nuclease free water and used immediately in PCR. Other samples were precipitated and air dried, then stored for 1 week at either 4°C or at RT. Lanes 1 – 3: dNTPs prepared fresh and applied immediately in PCR after resuspension in nuclease free water. Lanes 4 – 6: dNTPs stored in precipitated form at 4C for 1 week before resuspension in nuclease free water and application in PCR. Lanes 7 – 9: dNTPs stored in precipitate form at RT for 1 week before resuspension in nuclease free water and application in PCR. L: 1 kb plus ladder (NEB).

References

(1) Agarwal, R. P.; Robison, B.; Parks, R. E. Nucleoside Diphosphokinase from Human Erythrocytes. *Methods Enzymol*. **1978**, 51 (C), 376–386. https://doi.org/10.1016/S0076-6879(78)51051-3.

(2) Tan, Y. W.; Hanson, J. A.; Yang, H. Direct Mg2+ Binding Activates Adenylate Kinase from Escherichia Coli. *J. Biol. Chem.* **2009**, 284 (5), 3306–3313. https://doi.org/10.1074/jbc.M803658200.

(3) Oeschger, M. P. Guanylate Kinase from Escherichia Coli B. *Methods Enzymol.* **1978**, 51 (C), 473–482. https://doi.org/10.1016/S0076-6879(78)51065-3.

(4) Fehlau, M.; Kaspar, F.; Hellendahl, K. F.; Schollmeyer, J.; Neubauer, P.; Wagner, A. Modular Enzymatic Cascade Synthesis of Nucleotides Using a (d)ATP Regeneration System. *Front. Bioeng. Biotechnol.* **2020**, 8, 854. https://doi.org/10.3389/fbioe.2020.00854.

(5) Chen, P.; Liu, Z.; Liu, S.; Xie, Z.; Aimiuwu, J.; Pang, J.; Klisovic, R.; Blum, W.; Grever, M. R.; Marcucci, G.; et al. A LC-MS/MS Method for the Analysis of Intracellular Nucleoside Triphosphate Levels. *Pharm. Res.* **2009**, 26 (6), 1504. https://doi.org/10.1007/S11095-009-9863-9.
